# Supplementary material for: Enhanced polymer mechanical degradation through mechanochemically unveiled lactonization
Source: Nat Commun. 2020 Oct 5;11:4987. doi: 10.1038/s41467-020-18809-7 (PMC7536186; doi:10.1038/s41467-020-18809-7)
Supplement: Supplementary file 1 — Supplementary Information [file 41467_2020_18809_MOESM1_ESM.pdf]

Supporting Information

Enhanced Polymer Mechanical Degradation through Mechanochemically Unveiled Lactonization

Yangju Lin*, sTatiana B. Kouznetsova, Chia-Chih Chang, and Stephen L. Craig*
Department of Chemistry, Duke University, Durham, North Carolina 27708, United States

Contents

Supplementary Methods .... Error! Bookmark not defined.

Materials 3

Characterization 3

I. Synthesis 4

1. Overall synthetic scheme 4

2. Synthesis of small molecules 5

1) Synthesis of 1 5

2) Synthesis of 2 5

3) Synthesis of 3 6

4) Synthesis of 4 6

5) Synthesis of 5 6

3. Synthesis of polymers 7

1) Synthesis of P1 7

2) Synthesis of P2 7

4. Preparation of blended polymers 9

II. Sonication experiment 9

1. General sonication procedure 9

2. Analysis of mechanical activation 9

1) Ultrasonication of P2 9

2) Ultrasonication of P3 12

III. Extrusion study 15

IV. SMFS analysis 18

1. Determination of the thermal activation energy 18

2. SMFS curve analysis 20

3. Additional SMFS curves 22

V. CoGEF modeling 24

1. End-to-end distance modeling of ring closed BCOE 24

2. End-to-end distance modeling of ring opened BCOE 25

VI. NMR spectra 26

VII. Supplementary References 34

Supplementary Methods

Materials

Lab general solvents (hexane, ethyl acetate, dichloromethane, toluene, tetrahydrofuran, acetonitrile, methanol) were purchased from VWR or Sigma Aldrich. Tetrahydrophthalic anhydride (THPA), 2-butyne-1,4-diol, but-3-en-1-ol, benzophenone, *p*-toluenesulfonic acid (*p*TSA), 3,4-dihydro-2*H*-pyran, 9-oxabicyclo[6.1.0]non-4-ene, 4-(dimethylamino)pyridine (DMAP), N,N'-Diisopropylcarbodiimide (DIC), coumarin-3-carboxylic acid, polycaprolactone (PCL, 80 kDa) and Grubbs II catalyst were purchased from Sigma Aldrich, Alfa Aesar, Acros or TCI and used without further purification.

Characterization

^1H NMR spectra were collected on a Bruker Advance Neo-500 MHz multinuclear NMR spectrometer. Chemical shifts are provided in *ppm* (δ) and referenced to the residual ^1H peak at 7.26 ppm in $CDCl_3$. ^1H shifts are reported as chemical shift, multiplicity, coupling constant if applicable, and relative integral. Multiplicities are reported as: singlet (s), doublet (d), doublet of doublets (dd), doublet of triplets (dt), doublet of doublet of doublets (ddd), doublet of doublet of triplets (ddt), triplet (t), triplet of doublets (td), quartet (q), pentet (p), multiplet (m), or broad (br). Coupling constants (J) are reported in Hz. High-resolution mass spectra were collected on an Agilent LCMS-TOF-DART at Duke's Mass Spectrometry Facility.

Photoreaction was conducted using a photochemical reactor from The Southern New England Ultraviolet Company (Model #RPR-100, RPR2537A/254 nm bulb). Ultrasonication was performed with a Sonics VCX 750 generator using a 13 mm tip. Pulsed ultrasound (1s on, 1s off) was applied under N_2 atmosphere while cooled with an ice bath. Aliquot was taken from the solution and subjected to GPC and ^1H NMR analysis. Extrusion study was performed on a HAAKE ™ MiniCTW Micro-Conical Twin Screw Compounder from Thermo Scientific ™.

Gel permeation chromatography (GPC) was performed on two Agilent PLgel mixed-C columns (10^5 Å, 7.5x300 mm, 5 µm, part number PL1110-6500) using THF (stabilized with 100 ppm BHT) as the eluent. Molecular weights were calculated using a Wyatt Dawn EOS multi-angle light scattering (MALS) detector and Wyatt Optilab DSP Interferometric Refractometer (RI). The refractive index increment (dn/dc) values were determined by online calculation based on injections of known concentration and mass.

Sharp Microlever silicon probes (MSNL) and Silicon Nitride AFM Probes (PNP-DB) were correspondingly purchased from Bruker (Camarillo, CA) and NanoAndMore(Watsonville, CA). All of the SMFS studies were conducted at ambient temperature (~23 °C) using a homemade AFM, which was constructed using a Digital Instruments scanning head mounted on top of a piezoelectric positioner, similar to the one described in detail previously.[1-2] The AFM pulling experiments were conducted in a solution of toluene. The spring constant of each cantilever was calibrated in air, using the thermal noise method, based on the energy equipartition theorem as described previously.[1-2] Measurements were carried out in a closed fluid cell with a scanning set for a series of approaching/retracting cycles. Probes were prepared by immersing in piranha solution (H_2SO_4: H_2O_2 = 3:1) for 15 minutes at room temperature and then immersing in deionized water and dried by touching them against a borohydride. Silicon substrates were prepared by first allowing each to soak in hot piranha solution for 30 minutes and then washed with deionized water and dried under a stream of nitrogen. Caution should be used when handling piranha solution: it has been reported to detonate unexpectedly. The substrate and the cantilever were then placed in a UVO cleaner (ozone produced through UV light) for 15 minutes. After ozonolysis, the cantilever was mounted in the fluid cell. 20 µL of a 0.05-0.1 mg/mL polymer solution was added to the silicon substrate surface and allowed to dry. The silicon substrate was then placed on the piezoelectric stage of the AFM. Force curves were collected in dSPACE (dSPACE Inc. Wixom, MI) and analyzed using Matlab (The MathWorks, Inc., Natick, MA). All data were filtered during acquisition at 500 Hz. After acquisition, the data were calibrated and plotted by using homemade software written in Matlab language.

I. Synthesis

1. Overall synthetic scheme

Small molecule

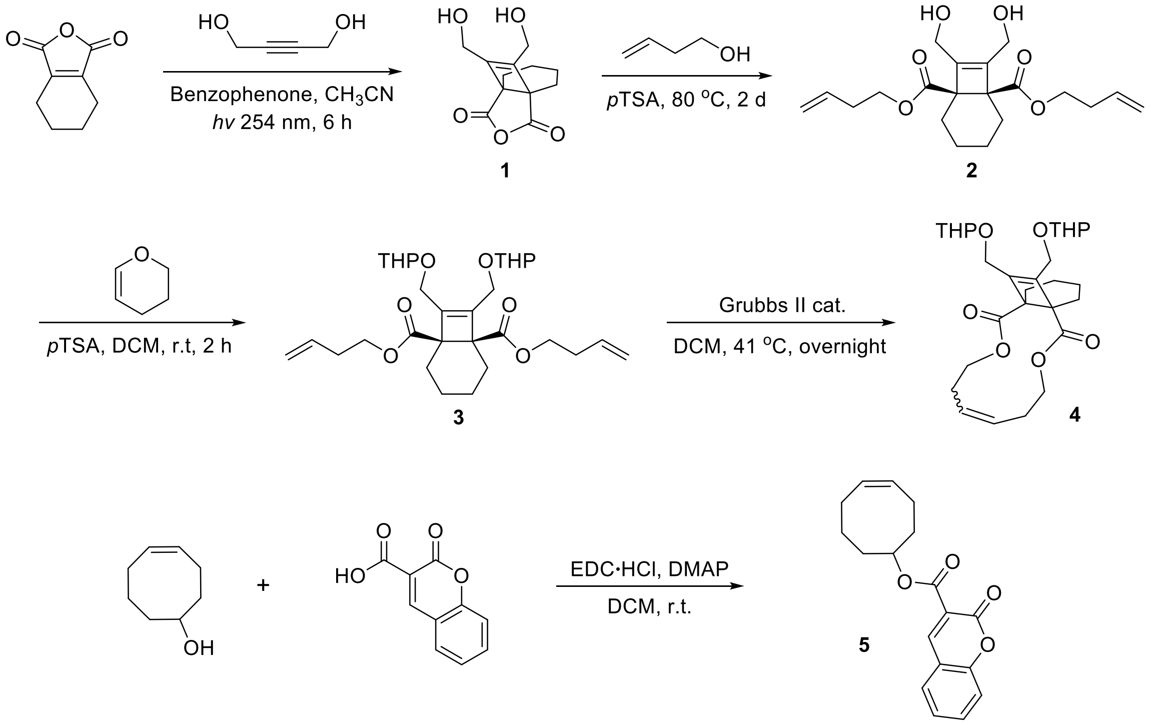

Polymer

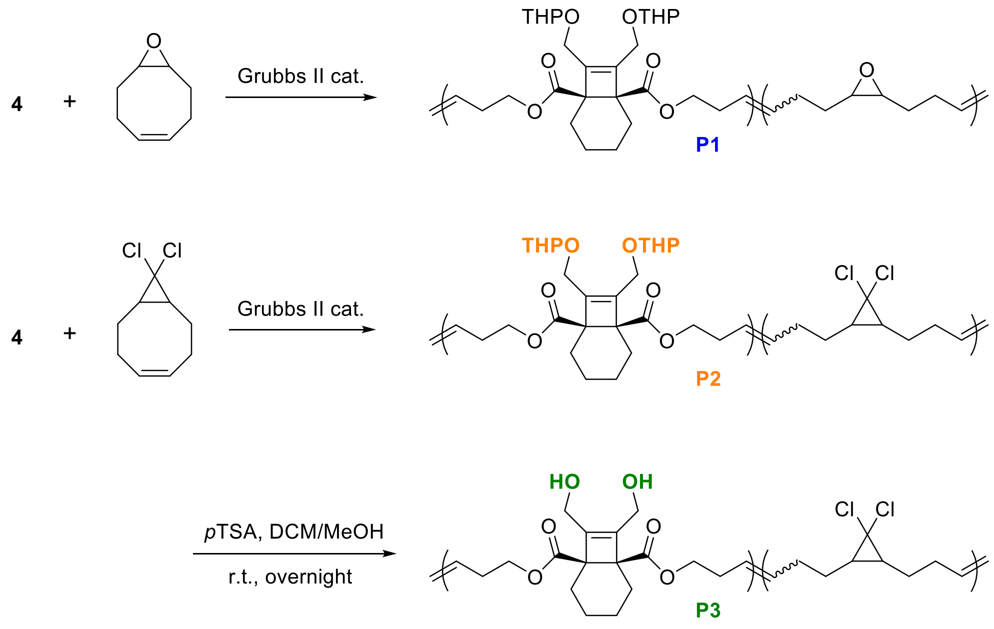

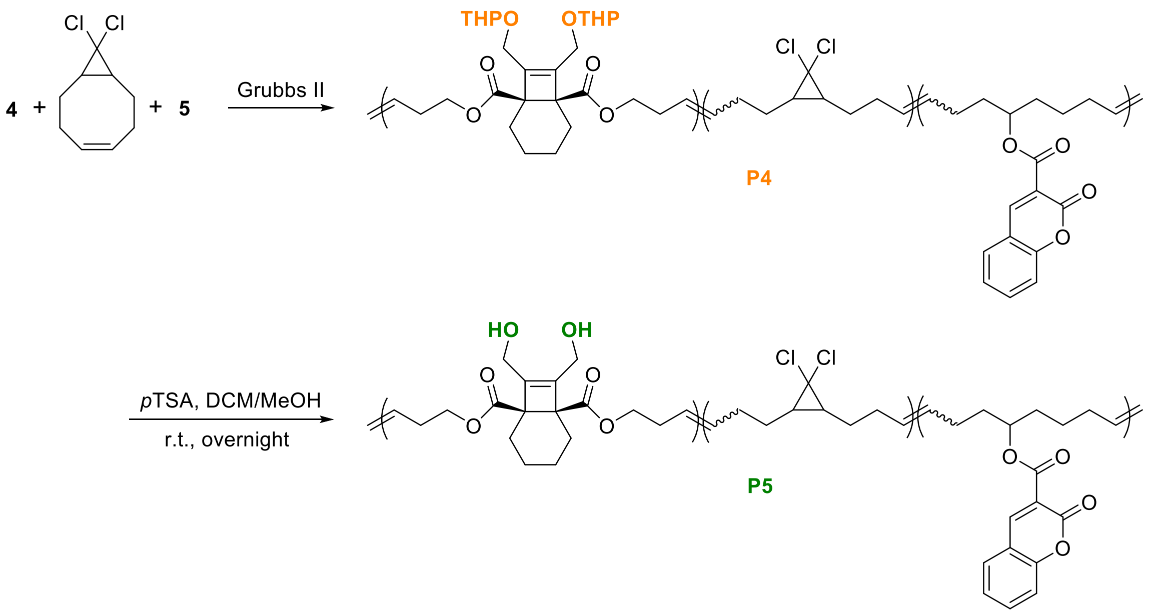

2. Synthesis of small molecules

1) Synthesis of **1**

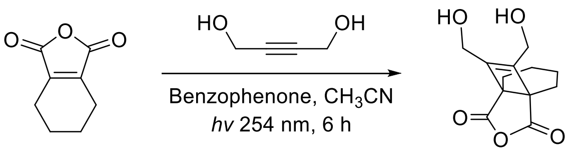

1

The synthesis of compound **1** was performed using procedures adapted from previous literature.[3-6]

To a 500 mL quartz flask, added tetrahydrophthalic anhydride (THPA) (2.28 g, 15 mmol), 2-butyne-1,4-diol (1.835 g, 22.5 mmol) and benzophenone (900 mg). 188 mL CH_3CN was then added to dissolve the compounds and the solution was sparged with N_2 for 10 min. The flask was capped and irradiated with UV 254 nm light for 6 h. The resulting pale-orange solution was condensed. Product was purified by chromatography using 0~70% EtOAc/hexane gradient eluent. Compound **1** was isolated as a pale-yellow solid (2.536 g, ^{1}H NMR indicates 1:1 molar ratio of product **1** to 2-butyne-1,4-diol, 52.2% yield). ^{1}H NMR (500 MHz, $CDCl_3$) δ: 4.31 (s, 4H), 2.14 – 1.91 (m, 4H), 1.72 – 1.59 (m, 2H), 1.59 – 1.45 (m, 2H). ^{13}C NMR (125 MHz, $CDCl_3$) ^{13}C NMR (125 MHz, $CDCl_3$) δ: 171.77, 145.08, 57.73, 51.27, 24.21, 19.45. HRMS-ESI (*m/z*): $[M+H]^+$ calculated for $C_{12}H_{15}O_5$, 239.09140; observed, 239.09204.

2) Synthesis of **2**

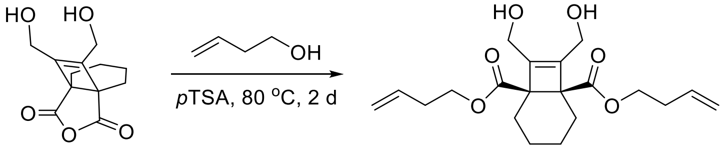

1 **2**

To a 50 mL round bottom flask, added compound **1** (1.19 g, 3.67 mmol compound **1**), *p*TSA (47 mg, 0.25 mmol) and 20 mL 3-buten-1-ol. The solution was then heated at 80 °C for 2d. The excess 3-buten-1-ol was

then distilled under reduced pressure. Resulting yellow viscous mixture was purified by chromatography (0~50% EtOAc/hexane) to give compound **2** as a pale-yellow oil (659 mg, 49.3%). ^{1}H NMR (500 MHz, $CDCl_3$) δ: 5.76 (ddt, *J* = 17.0, 10.2, 6.7 Hz, 2H), 5.18 – 5.03 (m, 4H), 4.32 – 4.17 (m, 4H), 4.08 (t, *J* = 6.7 Hz, 4H), 2.36 (qt, *J* = 6.7, 1.4 Hz, 4H), 2.21 – 2.06 (m, 2H), 1.87 – 1.76 (m, 2H), 1.69 – 1.59 (m, 2H), 1.56 – 1.44 (m, 2H). ^{13}C NMR (125 MHz, $CDCl_3$) δ: 173.75, 143.13, 134.08, 117.57, 64.14, 58.06, 55.98, 33.09, 25.18, 16.10. HRMS-ESI (*m/z*): $[M+H]^+$ calculated for $C_{20}H_{29}O_6$, 365.19587; observed, 365.19625.

3) Synthesis of **3**

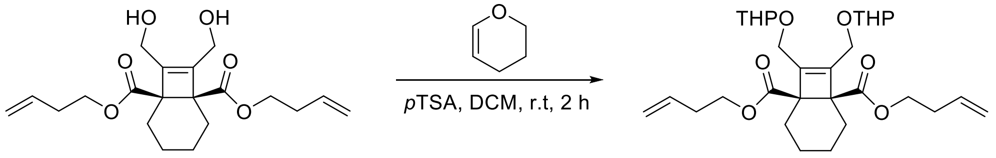

2 **3**

To a solution of compound **2** (364 mg, 1 mmol) in 10 mL DCM, added 3,4-dihydropyran (364 mg, 4 mmol) and *p*TSA (17 mg, 0.1 mmol). The solution was stirred at room temperature for 2 h. DCM was then removed and resulting oil was subjected to chromatography (0~30% EtOAC/hexane) to give compound **3** as a clear oil (322 mg, 60.5%). ^{1}H NMR (500 MHz, $CDCl_3$) δ: 5.77 (ddt, *J* = 17.0, 10.2, 6.9Hz, 2H), 5.15 – 5.00 (m, 4H), 4.64 (t, *J* = 3.3 Hz, 2H), 4.53 – 4.45 (m, 1H), 4.36 (dd, *J* = 13.2, 9.0 Hz, 1H), 4.26 – 4.18 (m, 1H), 4.16 – 4.11 (m, 1H), 4.11 – 3.99 (m, 4H), 3.87 – 3.74 (m, 2H), 3.55 – 3.46 (m, 2H), 2.35 (dddd, *J* = 9.0, 7.7, 4.7, 1.6 Hz, 2H), 2.18 – 2.07 (m, 2H), 1.93 – 1.83 (m, 2H), 1.83 – 1.72 (m, 2H), 1.72 – 1.45 (m, 14H).. ^{13}C NMR (125 MHz, $CDCl_3$) δ: 173.52, 141.58, 141.51, 134.25, 134.22, 117.30, 117.27, 98.13, 98.02, 63.80, 63.78, 63.75, 62.51, 62.47, 62.28, 62.26, 61.66, 61.62, 61.53, 61.51, 56.04, 56.01, 55.94, 33.17, 33.15, 31.74, 30.55, 30.53, 30.52, 26.13, 26.00, 25.92, 25.74, 25.63, 22.80, 19.10, 19.09, 19.07, 19.04, 16.13, 16.11, 16.08, 16.05, 14.27. HRMS-ESI (*m/z*): $[M+Na]^+$ calculated for $C_{30}H_{44}O_8Na$, 555.29284; observed, 555.29235.

4) Synthesis of **4**

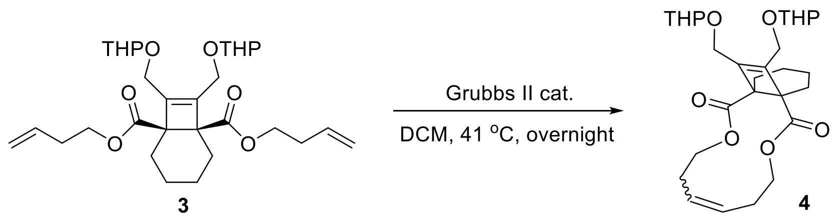

3 **4**

A solution of compound **3** (112 mg, 0.23 mmol) in 115 mL DCM was sparged with N_2 for 10 min, Grubbs II catalyst (20 mg, 0.023 mmol) was then added in one portion. The solution was heated at 41 °C for overnight. After the reaction completed, 0.2 mL of ethyl vinyl ether was added to quench the reaction and the solution was further stirred for 30 min. Resulting solution was rotavaped onto silica and purified by chromatography (0~30% EtOAc/hexane) to give compound **4** as an off-white solid (107 mg, 93%). Compound **4** was further recrystallized from hexane. ^{1}H NMR (500 MHz, $CDCl_3$) δ: 5.55 – 5.37 (m, 2H), 4.71 – 4.58 (m, 2H), 4.52 – 3.94 (m, 6H), 3.87 – 3.74 (m, 2H), 3.57 – 3.44 (m, 2H), 2.50 – 2.31 (m, 2H), 2.31 – 2.20 (m, 2H), 2.19 – 2.06 (m, 2H), 1.91 – 1.82 (m, 2H), 1.82 – 1.73 (m, 2H), 1.73 – 1.43 (m, 12H). ^{13}C NMR (125 MHz, $CDCl_3$) δ: 173.67, 173.52, 141.56, 141.22, 129.11, 129.08, 129.06, 128.61, 98.01, 97.95, 97.85, 63.42, 63.37, 63.31, 62.49, 62.23, 62.18, 61.65, 61.61, 61.31, 56.26, 56.22, 56.19, 31.15, 31.11, 31.08, 31.06, 30.52, 30.50, 27.54, 26.51, 26.28, 26.07, 25.78, 25.64, 19.09, 18.96, 18.93, 16.09, 16.05. HRMS-ESI (*m/z*): $[M+NH_4]^+$ calculated for $C_{28}H_{44}NO_8$, 522.30614; observed, 522.30747.

5) Synthesis of **5**

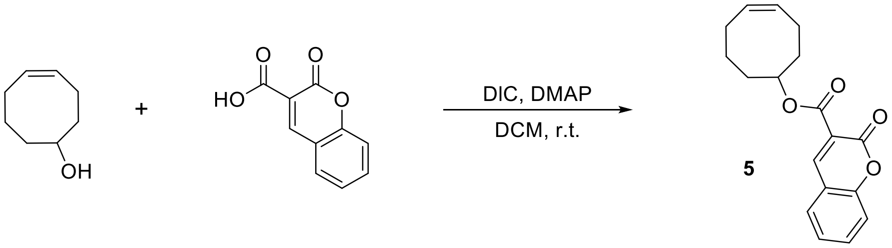

To a solution of (Z)-cyclooct-4-en-1-ol[7] (126 mg, 1 mmol) and coumarin-3-carboxylic acid (285 mg, 1.5 mmol) in 5 mL DCM, added DMAP (6.1 mg, 0.05 mmol). DIC (312 μL, 2 mmol) was then added dropwise. The resulting mixture was stirred at room temperature for 24h. After the reaction completed, insoluble solid was then filtered and the DCM was removed under reduced pressure. The crude was purified by flash chromatography (0~25% EtOAc/hexane gradient eluent). Compound was obtained as a pale yellow solid (284.7 mg, 95.5%). ^{1}H NMR (500 MHz, $CDCl_3$) δ: 8.43 (s, 1H), 7.73 – 7.54 (m, 2H), 7.39 – 7.28 (m, 2H), 5.80 – 5.59 (m, 2H), 5.09 (td, *J* = 9.4, 4.3 Hz, 1H), 2.41 (dddd, *J* = 14.5, 10.8, 8.4, 3.9 Hz, 1H), 2.28 – 2.10 (m, 3H), 2.09 – 1.63 (m, 6H). ^{13}C NMR (125 MHz, $CDCl_3$) δ: 162.49, 156.81, 155.25, 148.04, 134.27, 130.08, 129.68, 129.53, 124.88, 119.04, 118.05, 116.91, 77.75, 33.78, 33.61, 25.69, 24.96, 22.38. HRMS-ESI (*m/z*): $[M+Na]^+$ calculated for $C_{18}H_{18}NaO_4$, 321.10973; observed, 321.11068.

3. Synthesis of polymers

1) Synthesis of **P1**

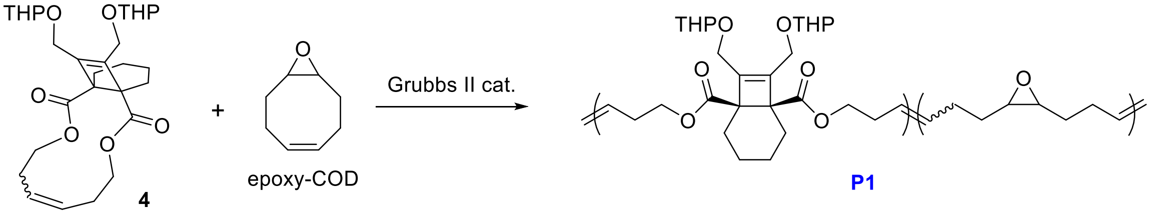

Monomer **4** (15.1 mg, 0.03 mmol) and 9-oxabicyclo[6.1.0]non-4-ene (epoxy-COD, 8.7 mg, 0.07 mmol) was weighted in a 10 mL frame-dried scintillation vial and kept under N_2. 0.1 mL of N_2 sparged Grubbs II catalyst solution (1.4 mg in 2 mL toluene) was added to the monomers. The polymerization was performed overnight, after which several drops of ethyl vinyl ether were added. The viscous solution was further stirred for 30min. After two rounds of precipitation from methanol, the resulting white polymer was dissolved in 1 mL DCM under stirring, and MeOH was added dropwise into the solution until it turned cloudy. The vial was capped and subjected to centrifuge at 4500 rpm for 10 min. The Upper clear solution was decanted and the polymer layer at the bottom was washed with MeOH and dried under high vacuum. This high molecular weight portion polymer (GPC-MALS: M_n = 118.6 kDa. $Đ_M$ = 1.48, dn/dc = 0.119) was further used for SMFS study.

2) Synthesis of **P2**

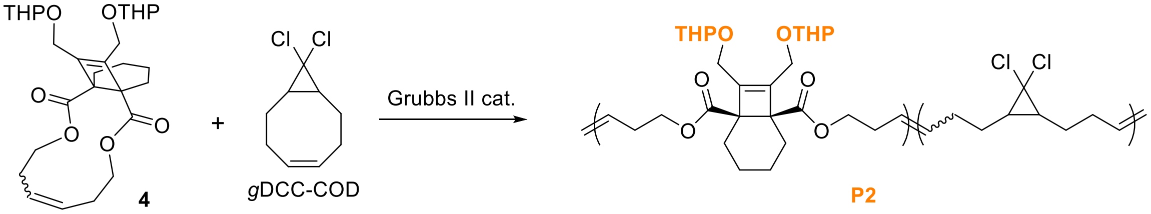

To a 10 mL frame-dried scintillation vial, added monomer **4** (25 mg, 0.05 mmol) and 9,9-dichlorobicyclo[6.1.0]non-4-ene[8-10] (gDCC-COD, 38.2 mg, 0.2 mmol). The vial was purged and kept under N_2. 0.25 mL of N_2 sparged Grubbs II catalyst solution (1.6 mg in 2 mL DCM) was added. After the polymerization was stirred for overnight, several drops of ethyl vinyl ether were added, and the solution was further stirred for 30 min to quench the catalyst. After

three rounds of precipitation from methanol, a white polymer was obtained (52 mg, 82%). ^{1}H NMR spectra indicated 14 mol% incorporation of monomer **4**. GPC-MALS: M_n = 128.4 kDa. $Đ_M$ = 1.39, dn/dc = 0.114.

3) Synthesis of **P3**

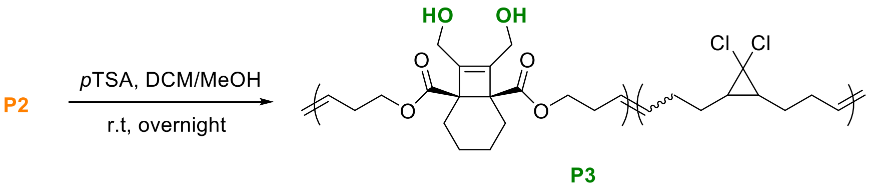

To a solution of 25 mg **P2** polymer in 1.5 mL DCM/MeOH (2:1), added 1 mg pTSA catalyst. After stirred for overnight, the solution was condensed and precipitated from MeOH. Obtained white polymer was dried under high vacuum (20 mg, 89%). ^{1}H NMR indicated selective removal of THP protecting group. The polymer was analyzed from GPC-MALS: M_n = 119.8 kDa. $Đ_M$ = 1.62, dn/dc = 0.124.

4) Synthesis of **P4**

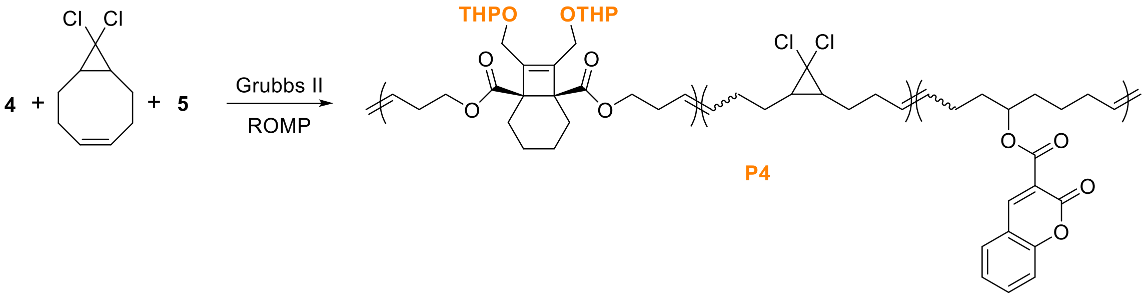

In a 10 mL frame-dried scintillation vial, monomer **4** (78 mg, 0.155 mmol), **5** (92 mg, 0.31 mmol) and *g*DCC-COD (59 mg, 0.2 mmol) were dissolved with 0.3 mL DCM. The vial was then purged with N_2, and 0.2 mL of N_2 sparged Grubbs II catalyst solution (2.6 mg in 0.8 mL DCM) was added. The polymerization was allowed to stir overnight, and several drops of ethyl vinyl ether were added to quench the polymerization. After stirred at room temperature for 30 min, polymer was precipitated from methanol. Two additional precipitation from methanol gave a white polymer (170 mg, 74%). ^{1}H NMR spectra indicated 5 mol% of **4** and 32 mol% of **5** incorporated into the polymer backbone. GPC-MALS: M_n = 68.6 kDa. $Đ_M$ = 1.71, dn/dc = 0.158.

5) Synthesis of **P5**

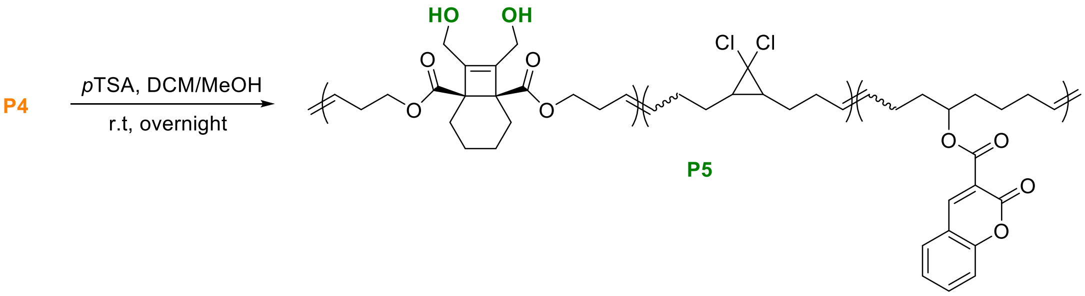

The procedure of preparing **P5** is similar to that of **P3**. ^{1}H NMR spectra indicated 5 mol% of BCOE and 32 mol% of **5** incorporated into the polymer backbone. GPC-MALS: M_n = 66.5 kDa. $Đ_M$ = 1.72, dn/dc = 0.158. The average number of BCOE along the polymer chain can be calculated as follows:

66500/(336*5%+191*63%+298*32%)*5% = 14

Hence, the average chain length between two adjacent BCEO mechanophore is: 66500/15 = 4433 ~ 4.4 kDa

4. Preparation of blended polymers

80 mg **P4** or **P5** polymer and 3.2 g commercial PCL polymer (80 kDa) were dissolved with 20 mL DCM, the viscous solution was vortexed and precipitated from methanol, and the resulting white polymer was dried under vacuum for 24 h. The polymer chunk was then cut into pieces using a scissors and further subjected to extrusion study.

II. Sonication experiment

1. General sonication procedure

A solution of polymer **P2** (1.2 mg mL^{-1}) and **P3** (1.0 mg/mL) in dry THF was transferred into a dry Suslick cell. The solution was sparged with N_2 for 10 min while cooled with ice bath. Pulsed ultrasound was applied (1s on, 1s off) at 30% amplitude. Aliquots of 0.8 mL sample at various sonication times (0, 5, 10, 20, 30, 45, 60 min) were took from the cell and analyzed by GPC. After GPC analysis, each remained samples was transferred into a 10 mL scintillation vial and condensed. Resulting polymer was further dried under high vacuum and then subjected to ^{1}H NMR analysis to quantify the amount of ring opened BCOE and *g*DCC mechanophores.

2. Analysis of mechanical activation

1) Ultrasonication of **P2**

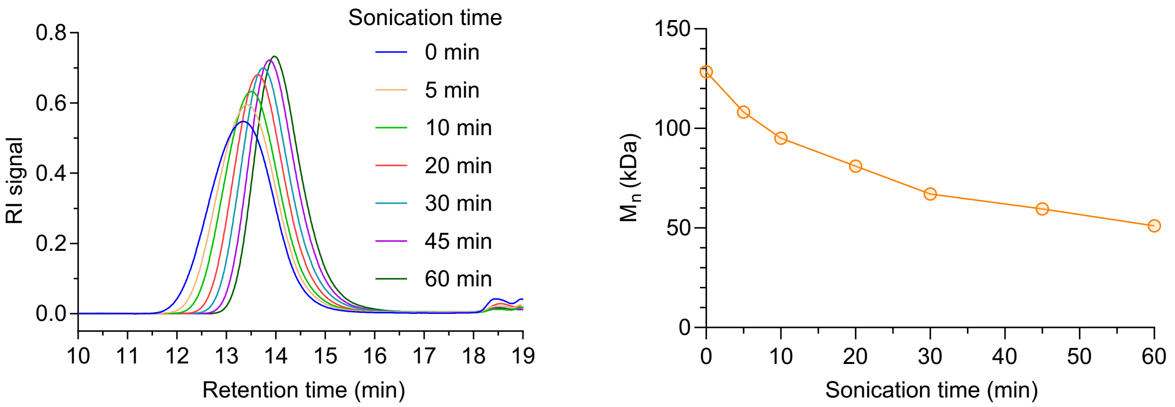

Supplementary Figure 1. GPC traces of **P2** polymer (left) and corresponding M_n evolution (right) at various sonication time. Each GPC sample was analyzed at the same concentration.

The number of chain scission cycle (SC) was calculated according to the following equation:

$$SC = \frac{\ln(M_n^{(0)}/M_n^{(t)})}{\ln 2}$$

$M_n^{(0)}$ is the initial molecular weight and $M_n^{(t)}$ is the sonicated molecular weight.

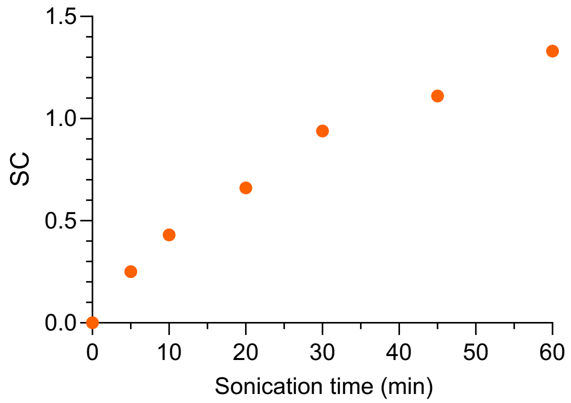

Supplementary Figure 2. Scission cycles of **P2** polymer at various sonication times.

Peak f in the ring opening product is used to quantify the extent of mechanophore activation. To verify the number of protons that contribute to the signal intensity of f, we noted that the activation percentage of BCOE:

$$\mathrm{RO(BCOE)}\ \% = \frac{\int \mathrm{H_f}/n}{\int \mathrm{H_d}/2*14\%} \times 100\% \qquad \text{(eq 1)}$$

Where $\int \mathrm{H_f}$ is the integration of peak f and n is the number of protons that are responsible for peak f, and $\int \mathrm{H_d}$ is the integration of 2 protons associated with the polymer backbone alkenes. 14% accounts for the stoichiometry of the mechanophore along the polymer backbone. Thus,

$$n = \mathrm{RO(BCOE)}\% * \frac{\int \mathrm{H_d}/2*14\%}{\int \mathrm{H_f}} \qquad \text{(eq 2)}$$

To determine RO(BCOE)%, we used the 60 min sonication sample. We used *p*TSA to cleave the THP protecting groups and then subjected the condensed mixture to ^{1}H NMR analysis. We obtained 53% activation (Figure 3). Plugging into equation 2, we calculate:

$$n = \frac{\int \mathrm{H_f}}{\mathrm{RO(BCOE)}\%*\int \mathrm{H_d}/2*14\%} = \frac{0.26}{53\%*3.91/2*14\%} = 1.8 \approx 2$$

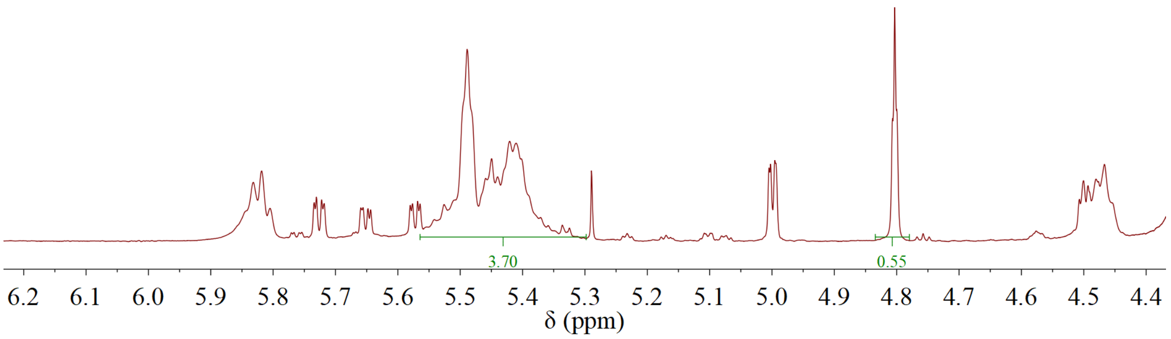

Supplementary Figure 3. ^{1}H NMR of **P2** polymer after 60 min sonication plus *p*TSA treatment for overnight. The percentage of generated lactone species equals to the fraction of mechanically activated BCOE: 0.55/2/3.70/0.14*100% = 53%. This number is similar to the result calculated using peak f in Figure 3 (48%). Therefore, peak f approximately corresponds to 2 protons in the ^{1}H NMR spectrum of Figure 4.

The activation of *g*DCC and BCOE mechanophores were analyzed from ^{1}H NMR spectrum. The ring-opening (RO) percentage of *g*DCC and BCOE are calculated from the following equations:

$$\mathrm{RO}(g\mathrm{DCC})\% = \frac{2*\int \mathrm{H_h}}{\int \mathrm{H_d}*86\%} \times 100\%; \quad \mathrm{RO(BCOE)}\ \% = \frac{\int \mathrm{H_f}}{\int \mathrm{H_d}*14\%} \times 100\%$$

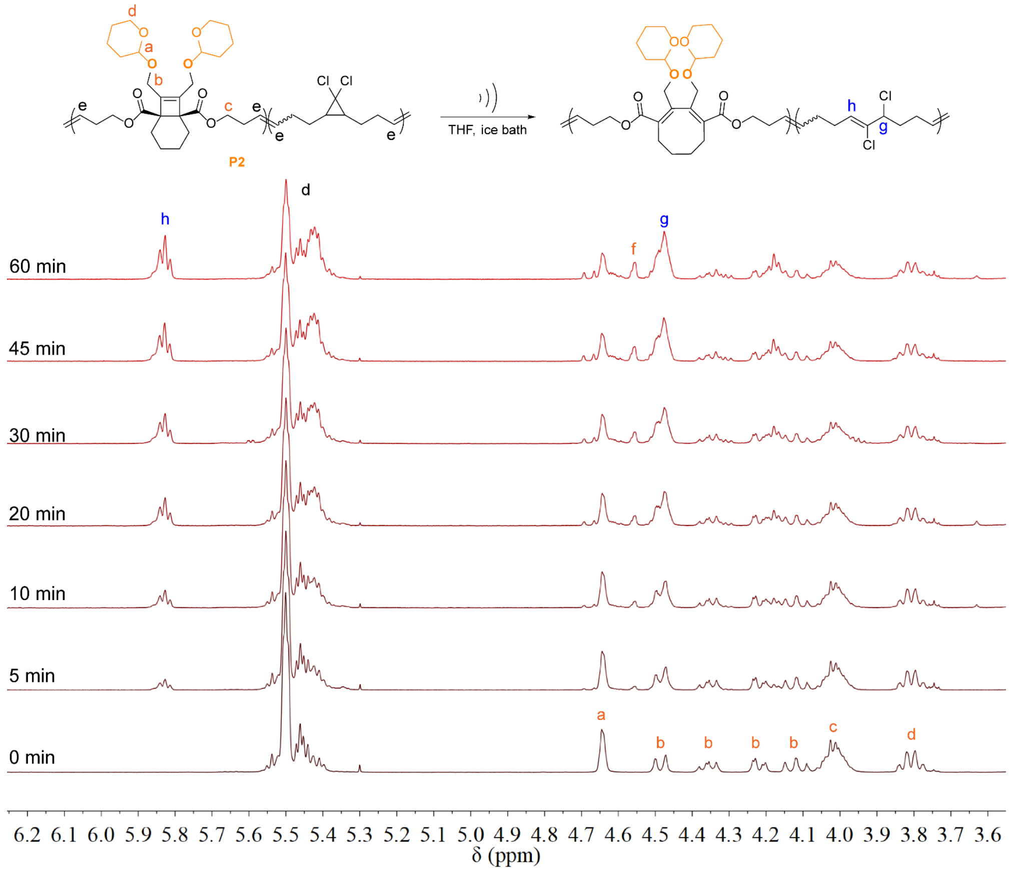

Supplementary Figure 4. Stack of ^{1}H-NMR ($CDCl_3$, 500 MHz) spectra of sonicated **P2** polymer at various sonication time.

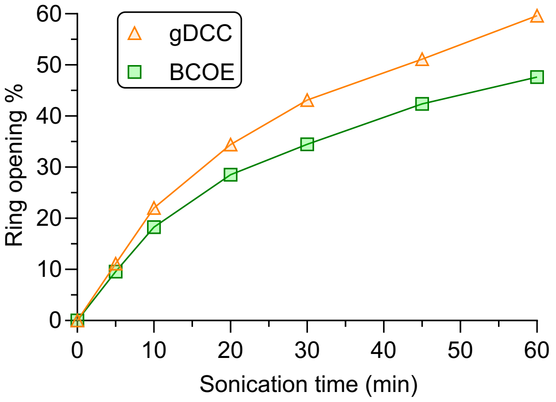

Supplementary Figure 5. Ring-opening percentage of *g*MCC and BCOE in polymer **P2** at various sonication time.

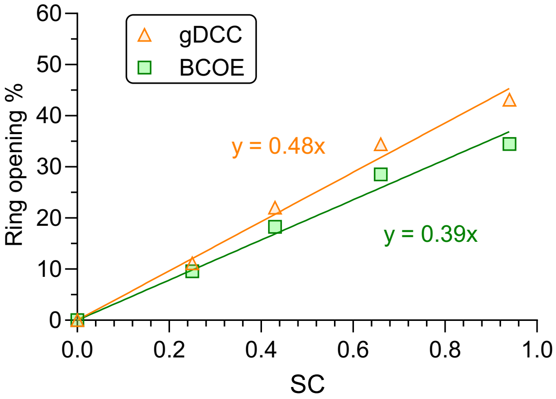

Supplementary Figure 6. Ring opening% of *g*DCC and BCOE vs. scission cycles. The slope (or Φ value) of linear fitting indicates the activation of mechanophore per chain scission. *g*DCC: $\Phi = 0.48$; BCOE: $\Phi = 0.39$.

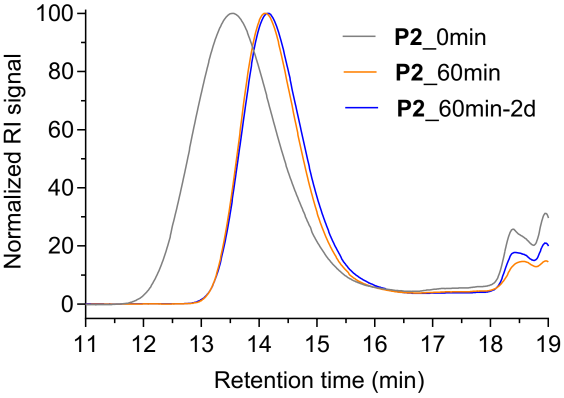

Supplementary Figure 7. M_n of a **P2** polymer decreased from 83.5 kDa ($Đ = 1.56$) to 43.8 kDa ($Đ = 1.22$) after 60 min ultrasonication treatment, and the M_n of sonicated **P2** remained nearly unchanged (42.5 kDa, $Đ = 1.27$) after 2d standing time. The peak intensity of each trace is normalized to 100.

2) Ultrasonication of **P3**

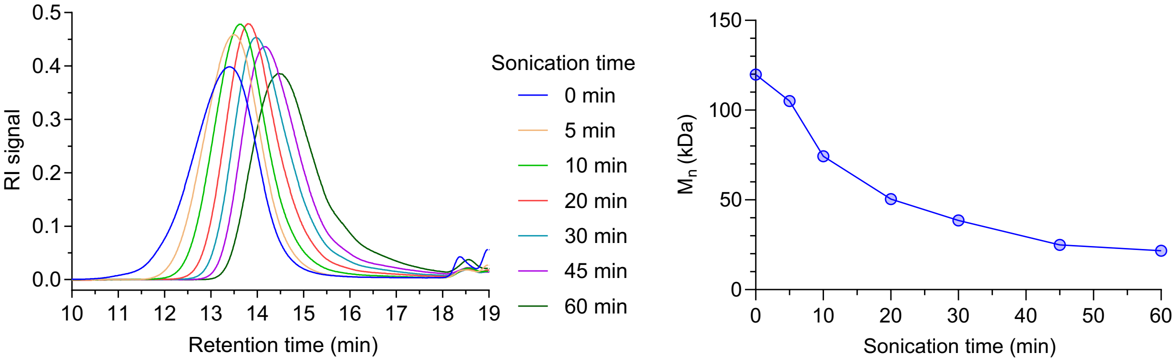

Supplementary Figure 8. GPC traces of **P3** polymer (left) and corresponding M_n evolution (right) at various sonication time. GPC analysis was performed immediately after sonication. Each GPC sample was analyzed at the same concentration.

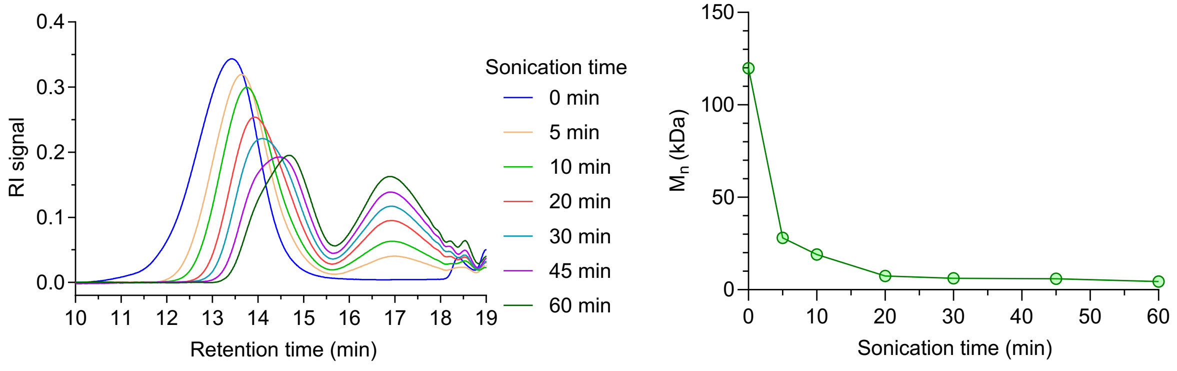

Supplementary Figure 9. GPC traces of **P3** polymer (left) and corresponding M_n evolution (right) at various sonication time. GPC analysis was performed after 17d of standing time. Each GPC sample was analyzed at the same concentration.

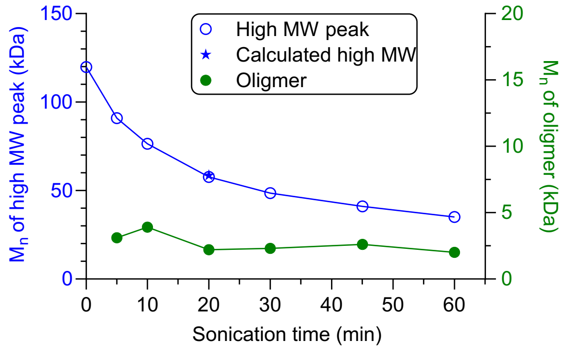

Supplementary Figure 10. Molecular weight (MW) analysis of **P3-17d** polymer. The M_n of high MW peak and oligomer peak were analyzed separately. The theoretical M_n of high MW fraction at 20 min sonication time was calculated assuming the same scission cycle as **P2** (SC = ln (128.4/81.1)/ln 2 = 0.66). The polymer has broken 2^{SC}-1 = 0.58 times. After one time of chain break, a 120 kDa polymer produces two 60 kDa daughter chains comprised of 39% activated BCOE (Figure S4) along the chain. According to reported method,[11] after removal of activated BCOE block, the theoretical M_n after 0.58 times of chain break is: M_n = (120*0.42+2*60*0.61*0.58)/(0.42+0.58*2) = 58.6 kDa.

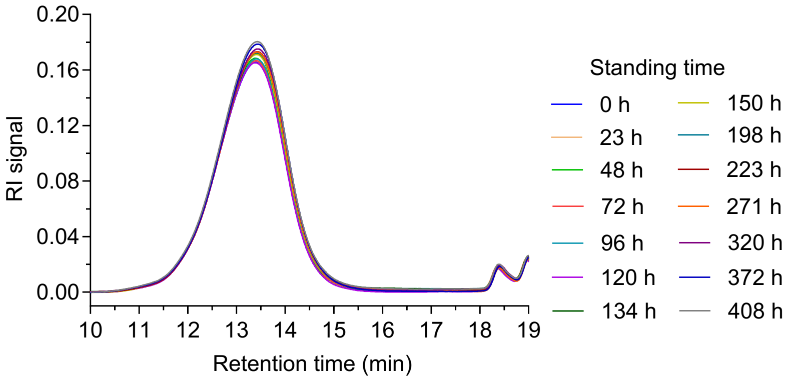

Supplementary Figure 11. GPC traces of pristine **P3** polymer at various standing time.

The fraction of lactone relative to incorporated BCOE mechanophore is calculated from the following equations:

$$\text{lactone\%} = \frac{\int H_d}{2 * \int H_c * 14\%} \times 100\%$$

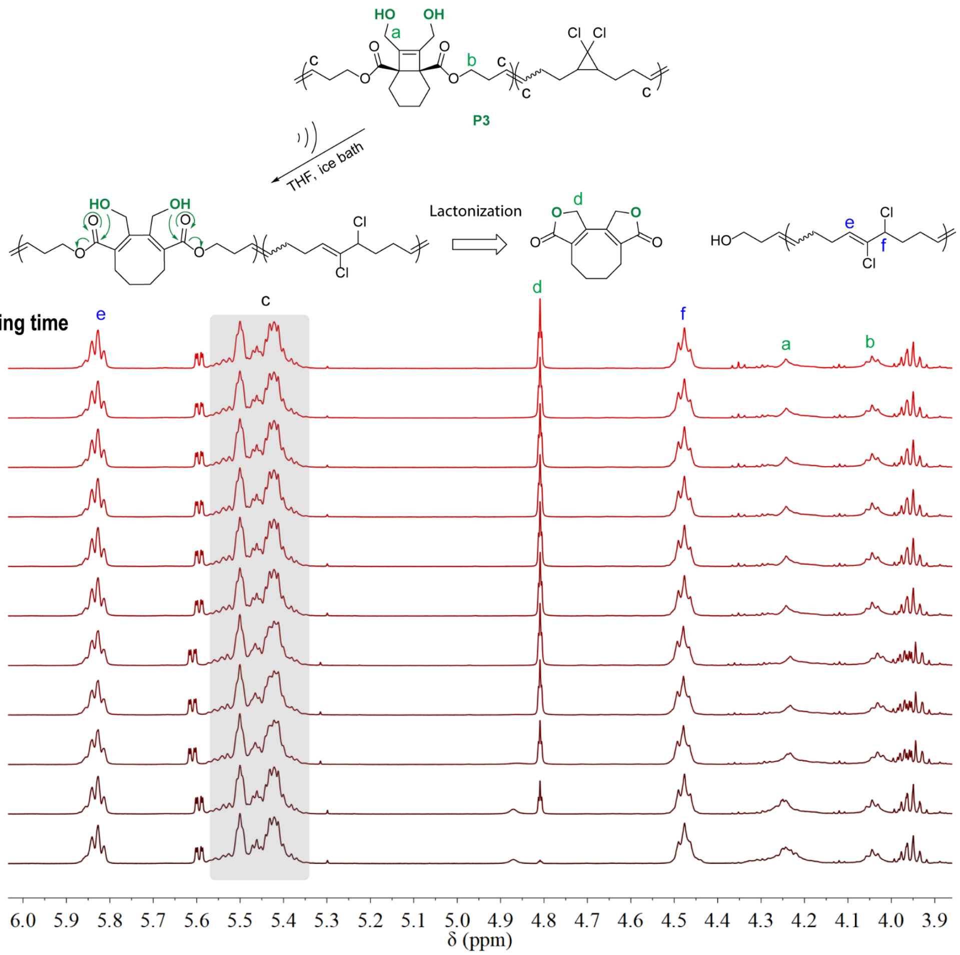

Supplementary Figure 12. ^{1}H-NMR (CDCl$_3$, 500 MHz) spectra stack of sonicated **P3** polymer at various standing time. **P3** polymer was subjected to ultrasonication for 60 min before ^{1}H NMR analysis.

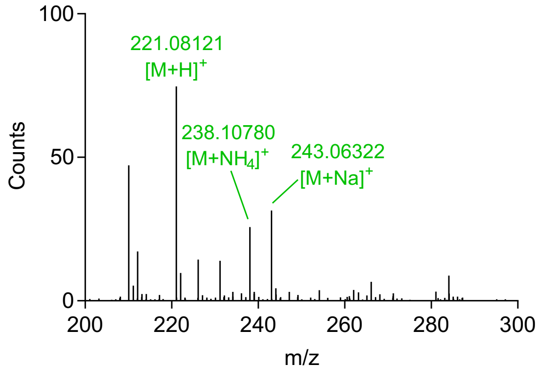

Supplementary Figure 13. HRMS analysis of **P3** polymer after 60 min sonication and further 20 days of standing.

III. Extrusion study

Extrusion study was performed on a HAAKE™ MiniCTW Micro-Conical Twin Screw Compounder. The compounder is comprised of a clamshell barrel with two conical screws and a recirculation pathway. The barrel was preheated to 65 °C and the screw rotation was set to 70 rpm. Polymer pieces (3.1g) were then added in portions using a mechanical plunger. The extrusion was performed for various of times. For P4, samples at 0, 5, 20, and 60 min extrusion were analyzed; P5 were sampled at 0, 6, 28, and 70 min extrusion.

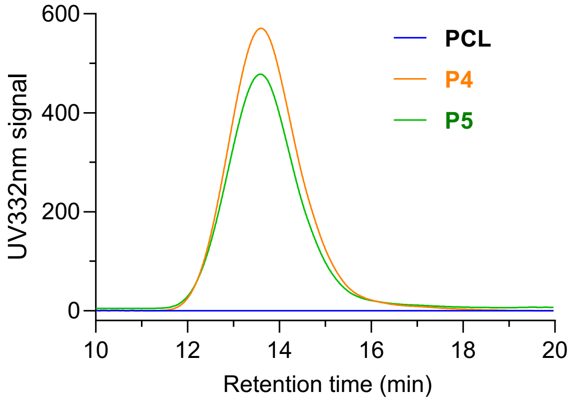

Supplementary Figure 14. UV 332nm signal of PCL, **P4** and **P5** polymers from GPC analysis (2 mg mL^{-1} in THF).

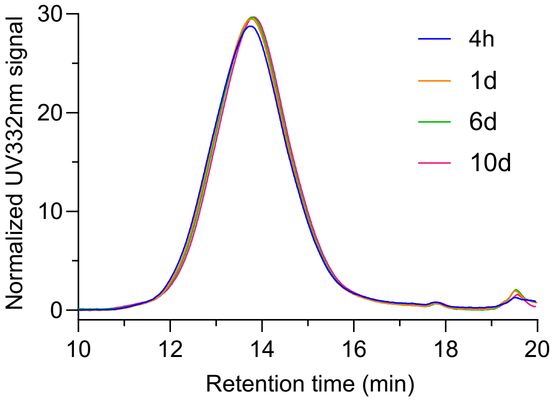

Supplementary Figure 15. Normalized GPC traces of **P4** polymer after 60min extrusion and further various standing times. The standing time is indicated in the legends. GPC traces are normalized to the area under peak.

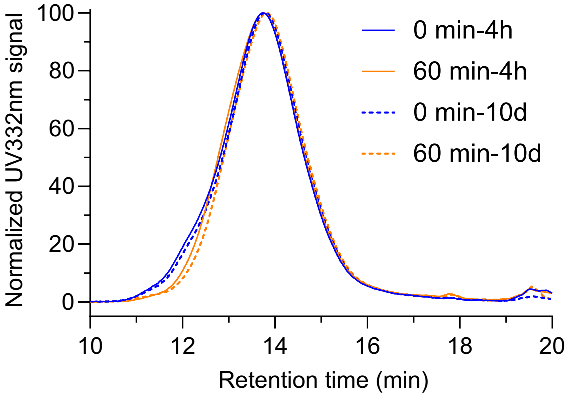

Supplementary Figure 16. Normalized GPC traces of **P4** polymer before and after 60 min extrusion with 4h and 10d standing time indicated in the legends. The signal from UV 332nm are normalized to the peak intensity.

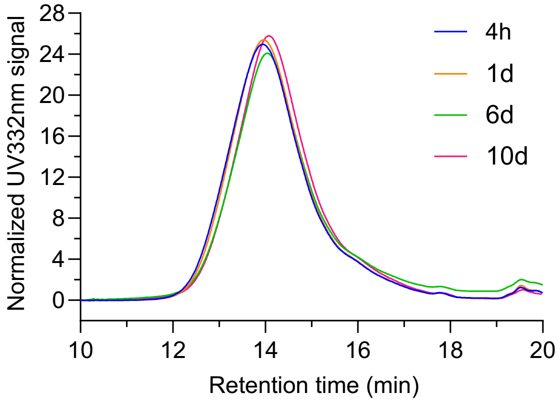

Supplementary Figure 17. Normalized GPC traces of **P5** polymer after 70 min extrusion and further various standing time. The standing time is indicated in the legends. GPC traces are normalized to the area under peak.

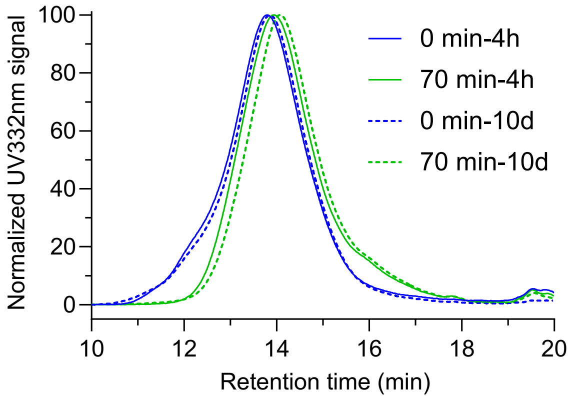

Supplementary Figure 18. Normalized GPC traces of **P5** polymer before and after 70 min extrusion with 4h or 10d standing time indicated in the legends. The signal from UV 332nm are normalized to the peak intensity.

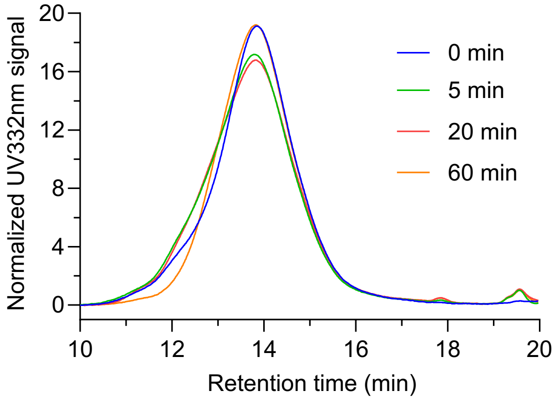

Supplementary Figure 19. Normalized GPC traces of **P4** polymer after different extrusion times plus 10 d standing time. The extrusion time is indicated in the legends. GPC traces are normalized to the area under peak.

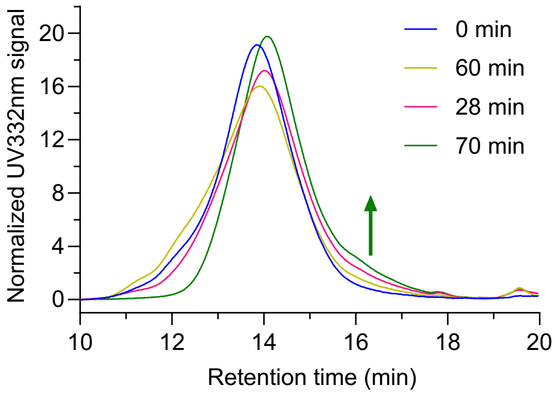

Supplementary Figure 20. Normalized GPC traces of **P5** polymer after different extrusion times plus 10 d standing time. The extrusion time is indicated in the legends. GPC traces are normalized to the area under peak.

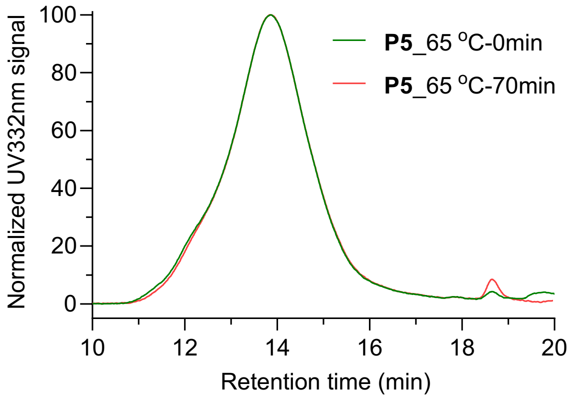

Supplementary Figure 21. Thermal control experiment. Overlay of normalized GPC traces of **P5** polymer before (green) and after (red) 70 min heating at 65 °C. The signal from UV 332nm are normalized to the peak intensity.

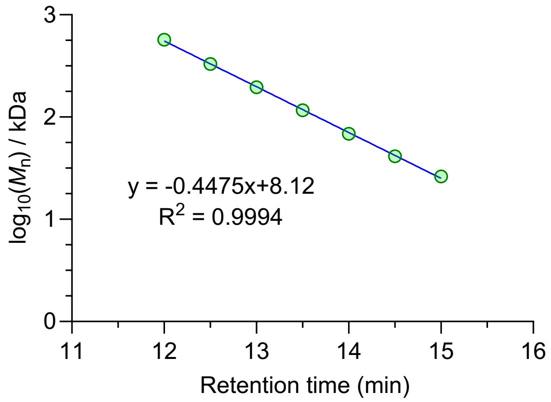

Supplementary Figure 22. The M_n corresponding to various retention time in **P5** polymer were retrieved from GPC-MALS analysis and plotted to give the calibration curve. Extrapolation of the calibration curve to 16 min and 17 min retention time gives M_n of 8.9 kDa and 3.2 kDa, respectively.

IV. SMFS analysis

1. Determination of the thermal activation energy

In a 25 mL dry Shlenk flask, 20 mg of BCOE diene derivative **3** was dissolved in 2 mL diphenyl ether. The flask was then heated to 150 °C under N_2. Aliquots of 0.1 mL solution at time point (0, 30, 60, 160, 270, 360 min) was took from the flask and dilute with $CDCl_3$ for ^{1}H NMR analysis. Note: Due to the more strained 8-member ring in the ring opened product and presence of THP protecting groups, the ring opened (RO) compound **3** can undergo reversible ring closing to give pristine ring closed (RC) form without producing lactone.[12-13]

The percentage of ring closed from in the system can be calculated using the following equation:

$$\mathrm{RC}\,\% = \frac{\int \mathrm{H_a} + \int \mathrm{H_b}}{\int \mathrm{H_c}} \times 100\%$$

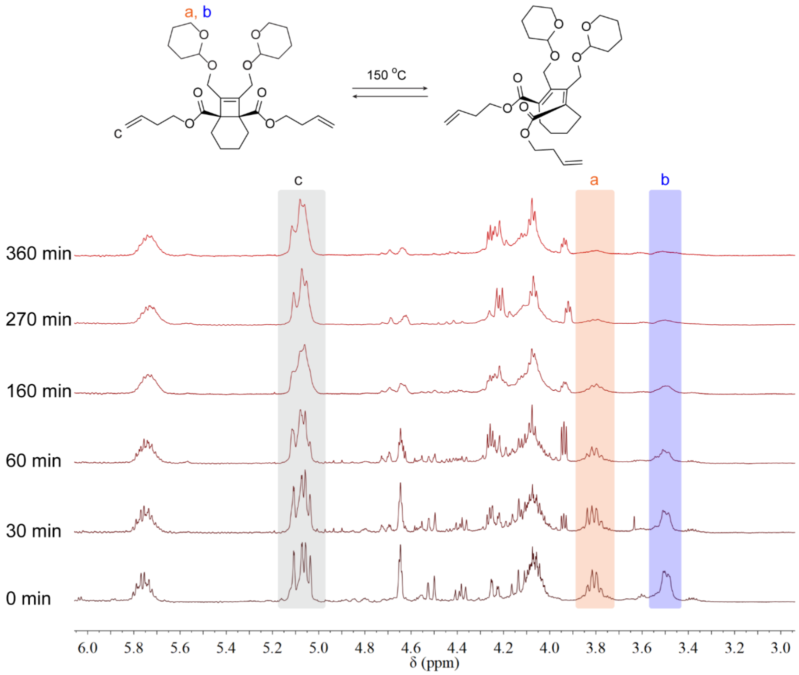

Supplementary Figure 23. ^{1}H NMR ($CDCl_3$, 500 MHz) spectra stack of compound **3** in diphenyl ether after heated at 150 °C for various time.

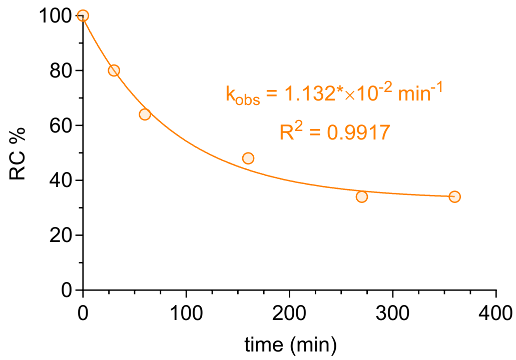

Supplementary Figure 24. Percentage of ring closed form over time at 150 °C in diphenyl ether.

The equilibrium at 150 °C can be estimated from the plateau (33%) of fitting:

$$K_{eq} = \frac{k_1}{\mathrm{k}_{-1}} = \frac{[\mathrm{RO}]}{[\mathrm{RC}]} \approx 2$$

The observed rate constants is: $k_{obs} = k_1 - k_{-1} = 1.132\times10^{-2}$ min^{-1}

Therefore, the ring-opening rate constant is: $k_1 = 2k_{-1} = 2k_{obs} = 2.264\times10^{-2}$ min^{-1} = 3.77×10^{-4} s^{-1}

According to the transition state theory, the thermal activation energy can be estimated:

$$\Delta G^{\ddagger} = -RT\ln\frac{k_1 h}{k_B T} = -8.314\times423\times\ln\frac{3.77\times10^{-4}\times6.626\times10^{-34}}{1.38\times10^{-24}\times423}\ \text{J/mol} = 132550\ \text{J/mol} = 31.7\ \text{kcal/mol}$$

Mechanically induced disrotatory ring opening of BCOE is a forbidden pathway. Hence, the activation energy of forbidden reaction would be at least 4 kcal/mol more than thermally allowed path way.[14] The activation energy of disrotatory ring opening of BCOE is:

$$\Delta G^{\ddagger} > 31.7 + 4 \text{ kcal/mol} = 35.7 \text{ kcal/mol}$$

2. SMFS curve analysis

Force-extension curves of polymer **P1** were analyzed using method reported previously.[15] Pre- and post- transition force curves were fitted with extended freely jointed chain (FJC) model to give the contour lengths of polymers before and after transition. Further analysis with Bell-Evans (BE) or Cusp models provided $\Delta x^{\ddagger}$ information.

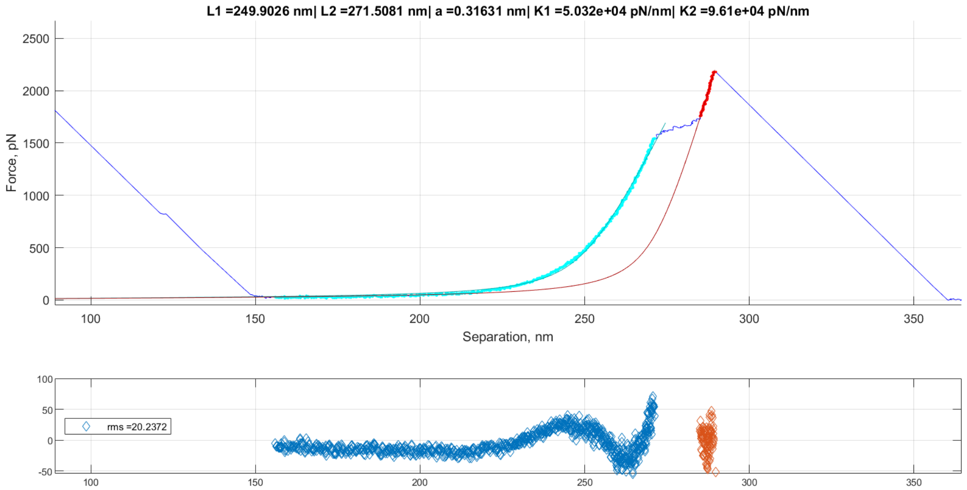

Supplementary Figure 25. Fitting of pre and post transition in force-extension curve with extended FJC model. The contour before and after transition are obtained: before, L_i = 249.9 nm; after, L_f = 271.5 nm.

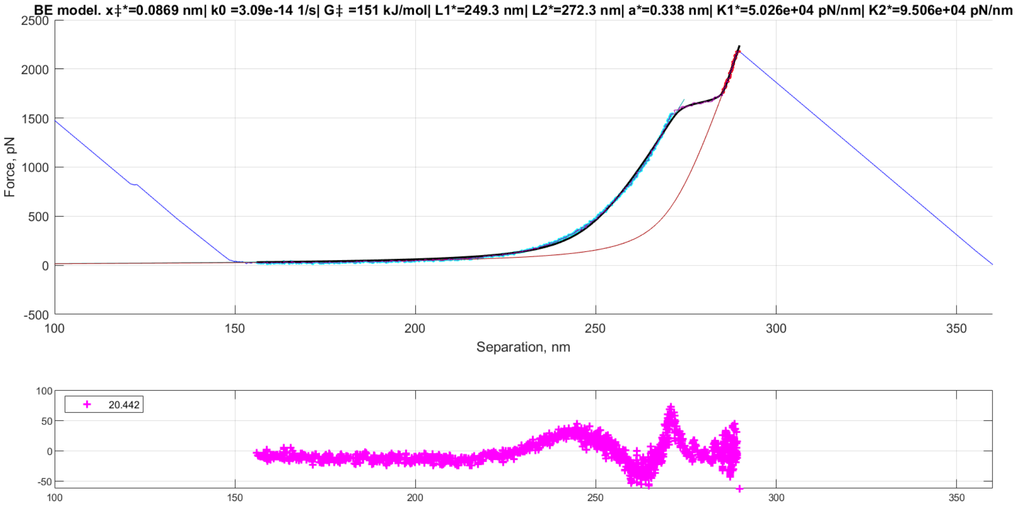

Supplementary Figure 26. Fitting of force-extension curve with BE model. Mechanical coupling $\Delta x^{\ddagger}$ = 0.87 Å.

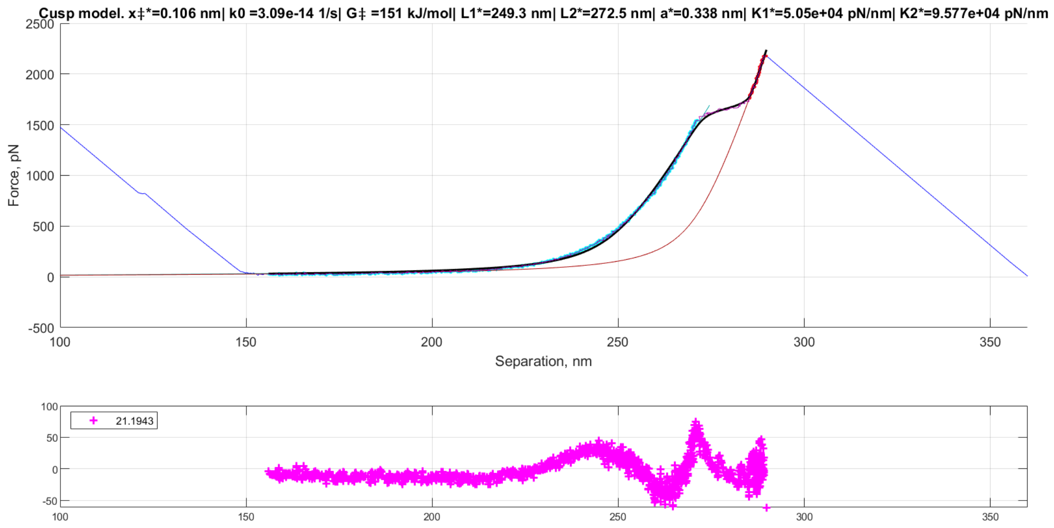

Supplementary Figure 27. Fitting of force-extension curve with Cusp model. Mechanical coupling $\Delta x^{‡} = 1.06$ Å.

Using reported method,[16] the BCOE ring-opening rate corresponding to different force values obtained from the surviving probability at the characteristic transition. The survival probability was calculated using the following equation:

$$S(F) = \frac{L_2 - L(F)}{L_2 - L_1}$$

L_1 and L_2 are the polymer contour lengths before and after the transition; L(F) is the polymer total length during the transition.

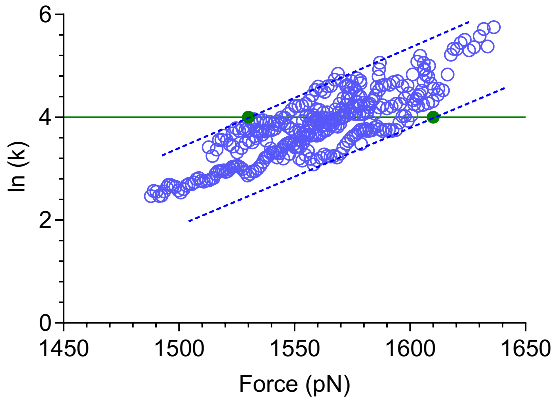

Supplementary Figure 28. Overlay of rate vs. force plots obtained from analysis of seven SMFS curves. The dotted blue lines empirically describe the width of rate-force relationships and intercept the line $y = 4$ at two green solid dots (x = 1530 and 1610 pN). The distance between two green dots is the force range (1570 ± 40 pN) required to achieve BCOE ring opening at the rate of $k = 55$ s^{-1}.

3. Additional SMFS curves

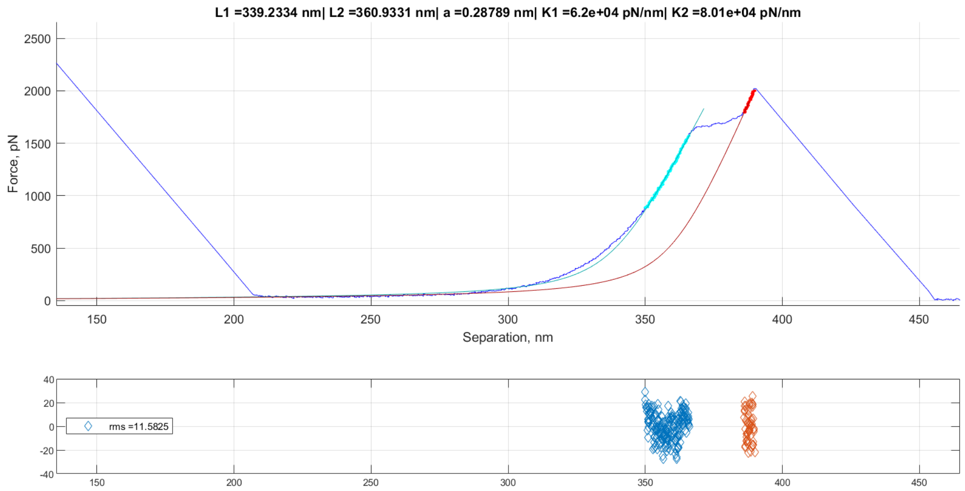

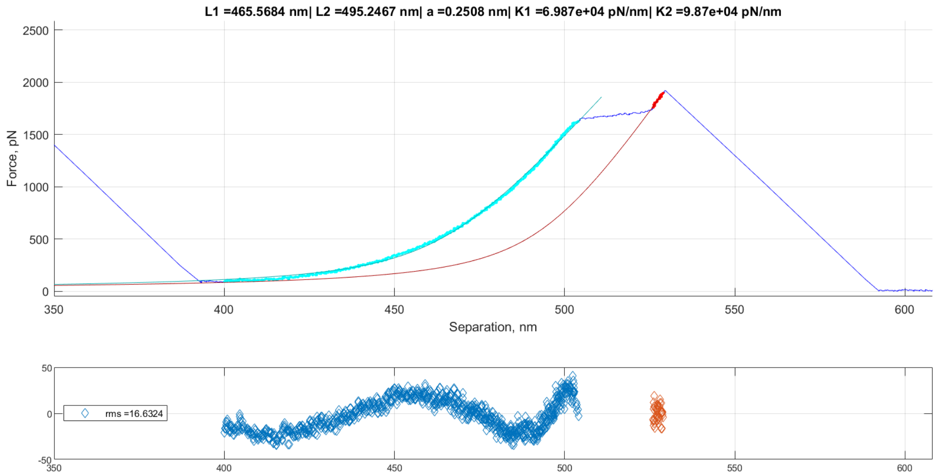

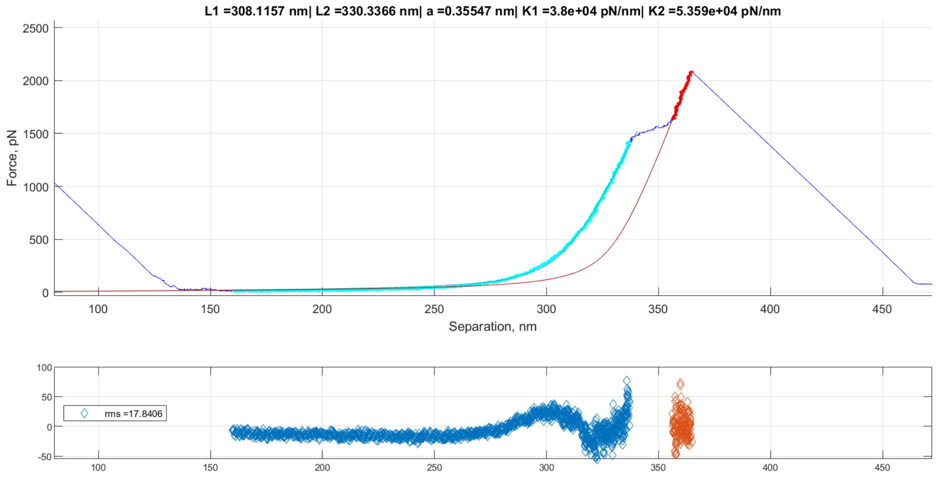

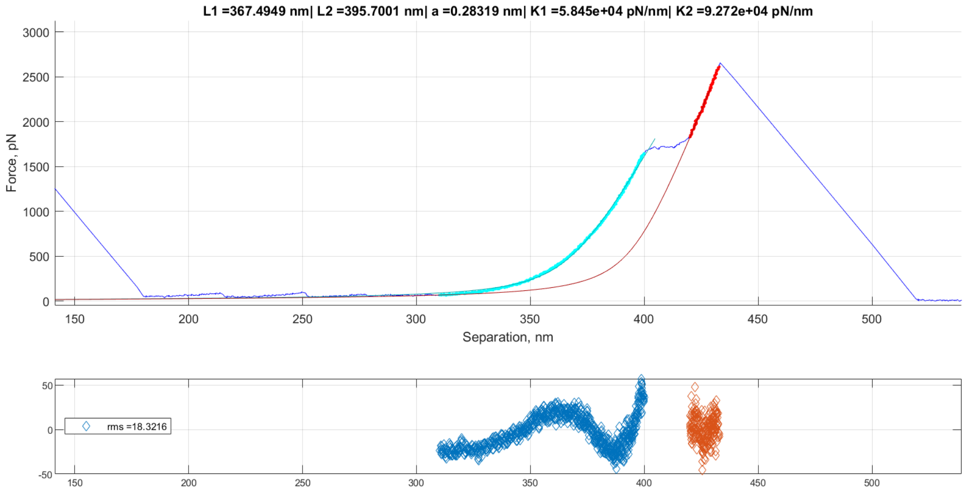
L1 =367.4949 nm| L2 =395.7001 nm| a =0.28319 nm| K1 =5.845e+04 pN/nm| K2 =9.272e+04 pN/nm
Force, pN
Separation, nm
rms =18.3216

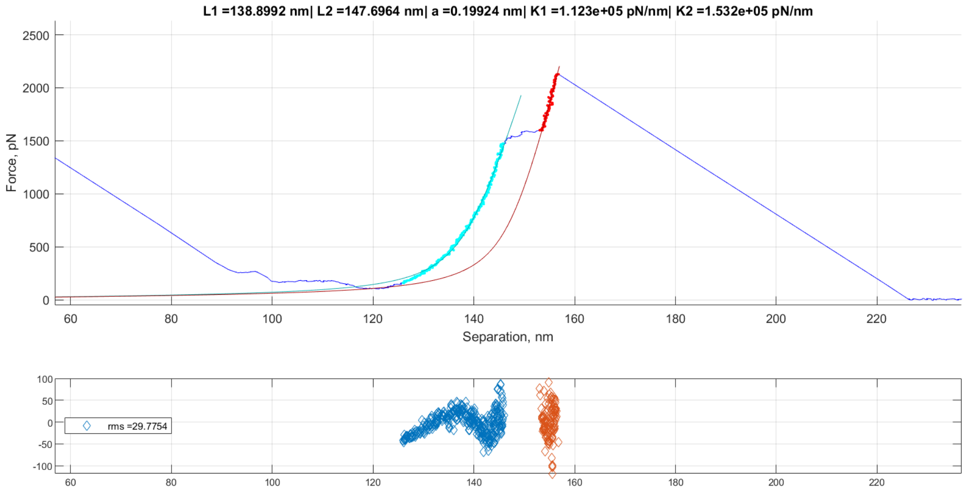
L1 =138.8992 nm| L2 =147.6964 nm| a =0.19924 nm| K1 =1.123e+05 pN/nm| K2 =1.532e+05 pN/nm
Force, pN
Separation, nm
rms =29.7754

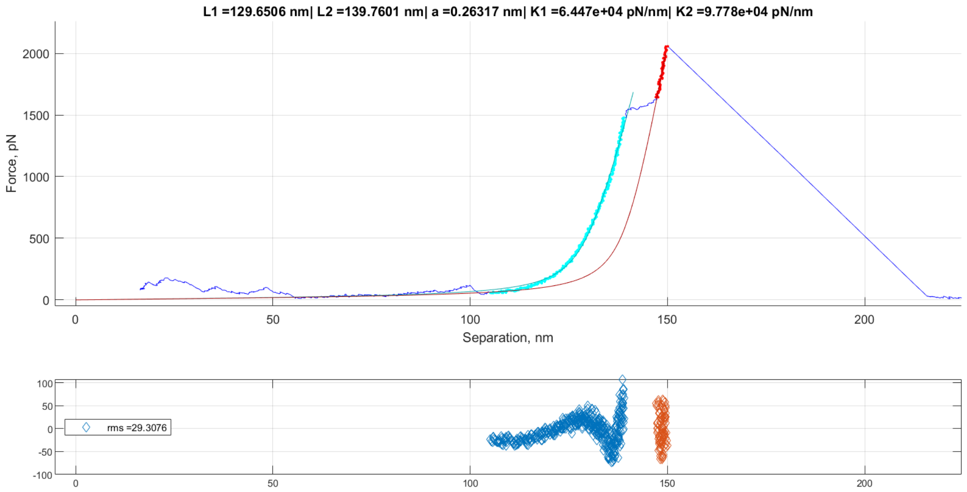
L1 =129.6506 nm| L2 =139.7601 nm| a =0.26317 nm| K1 =6.447e+04 pN/nm| K2 =9.778e+04 pN/nm
Force, pN
Separation, nm
rms =29.3076

Supplementary Table 1. List of results from SMFS curves analysis

#	L_i nm	L_f nm	L_f/L_i	Kuhn length (nm)	K_1 (pN/nm)	K_2 (pN/nm)	$\Delta x^{\ddagger}$		F* (pN)
							BE	Cusp	
1	99.5	105.0	1.056	0.225	7.51×10^4	8.31×10^4	0.94	1.15	1539
2	186.8	190.4	1.020	0.237	7.43×10^4	6.50×10^4	0.94	1.15	1557
3	198.4	209.7	1.057	0.279	6.45×10^4	8.19×10^4	0.91	1.11	1594
4	308.1	330.3	1.072	0.355	3.80×10^4	5.36×10^4	0.92	1.13	1529
5	339.2	360.9	1.064	0.288	6.20×10^4	8.01×10^4	0.84	1.03	1690
6	249.9	271.5	1.086	0.316	5.03×10^4	9.61×10^4	0.87	1.06	1645
7	465.6	495.2	1.064	0.251	6.99×10^4	9.87×10^4	0.84	1.03	1690
8	367.5	395.7	1.077	0.283	5.85×10^4	9.27×10^4	0.82	1.01	1716
9	138.9	147.7	1.063	0.199	1.12×10^4	1.53×10^4	0.93	1.14	1566
10	265.6	280.5	1.056	0.298	5.57×10^4	8.49×10^4	0.85	1.04	1704
11	129.6	139.8	1.078	0.263	6.45×10^4	9.78×10^4	0.92	1.13	1570
12	89.6	95.5	1.066	0.308	5.46×10^4	7.48×10^4	0.90	1.10	1609
Avg.			1.063				0.89	1.09	1617
SD.			0.017				0.04	0.05	68
**Report**			**1.063 ±0.017**				**0.89 ±0.04**	**1.09 ±0.05**	**1620 ±70**

V. CoGEF modeling

CoGEF modeling of BCOE repeating unit and corresponding forbidden ring opening product were performed on Spartan'16 V2.0.7 version at Molecular Mechanics/MMFF theory level. The distance of chain-end carbons was constrained and relaxed with step interval of 0.1 Å. The obtained energy at each relaxed step was plotted as a function of the end-to-end distance of repeating unit, which is indicated at the distance between the carbon atoms labeled with orange dots in the chemical structures. Further quadratic fitting and subsequent analysis from the first derivative gave force vs. extension relation, from which the contour length (x_0) at zero force can be extrapolated.

1. End-to-end distance modeling of ring closed BCOE

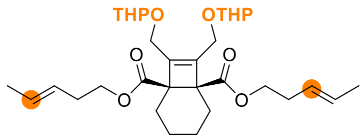

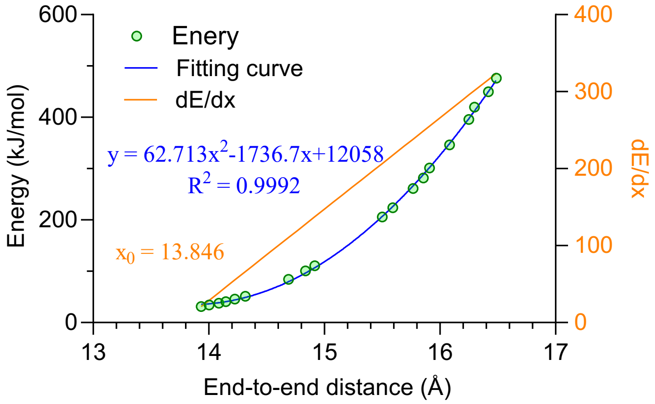

Supplementary Figure 29. Quadratic fit of energy vs. distance curve (blue); force vs. distance plot (orange). End-to-end distance of ring closed BCOE: x_0 = 13.846 Å.

2. End-to-end distance modeling of ring opened BCOE

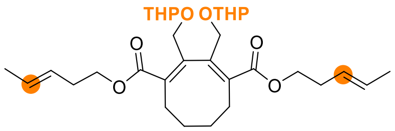

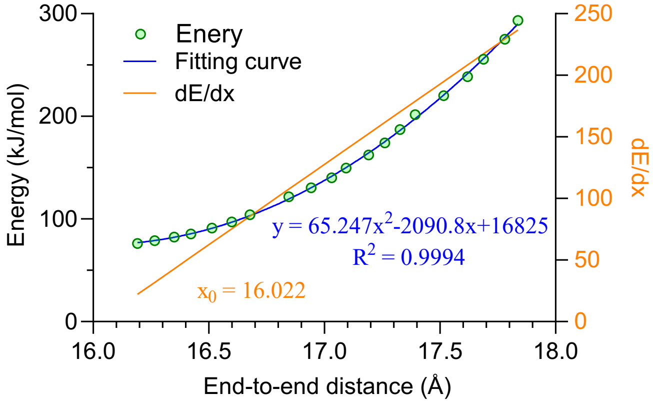

Supplementary Figure 30. Quadratic fit of energy vs. distance curve (blue); force vs. distance plot (orange). End-to-end distance of ring closed BCOE: x_0 = 16.022 Å.

The ratio of final contour length (L_f) to initial contour length (L_i) can be calculated:

$$\frac{L_f}{L_i} = \frac{x \times l_f + (1\text{-}x) \times l_{COD}}{x \times l_i + (1\text{-}x) \times l_{COD}} = \frac{0.26 \times 16.022 + 0.74 \times 9.366}{0.26 \times 13.846 + 0.74 \times 9.366} = 1.054$$

x is the incorporation percentage of BCOE; l_{COD} is the contour length of epoxy-COD repeating unit.[17]

VI. NMR spectra

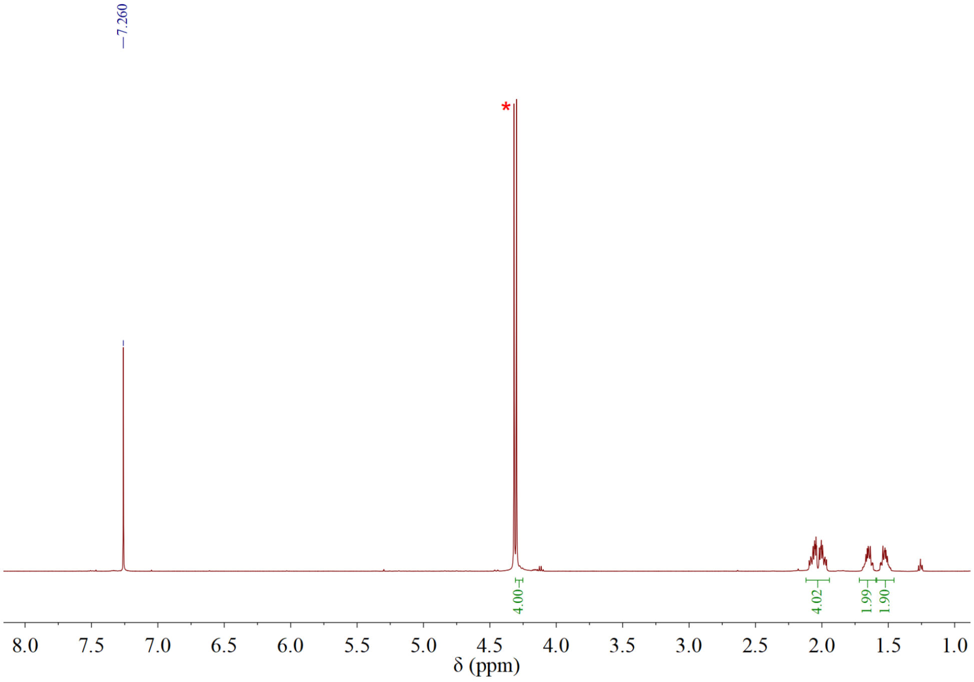

Supplementary Figure 31. ^{1}H NMR ($CDCl_3$, 500 MHz) spectrum of compound **1**. "*" labeled peak is from unreacted but-2-yne-1,4-diol.

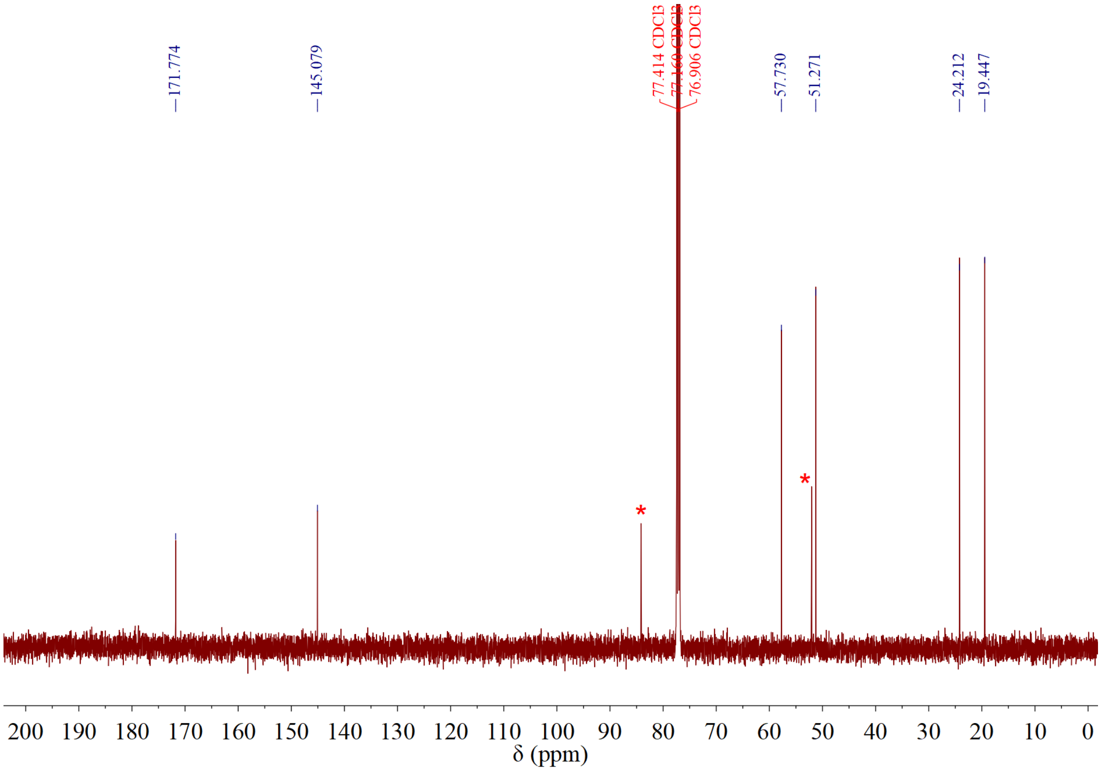

Supplementary Figure 32. ^{13}C NMR ($CDCl_3$, 125 MHz) spectrum of compound **1**. "*" labeled peaks are from unreacted but-2-yne-1,4-diol.

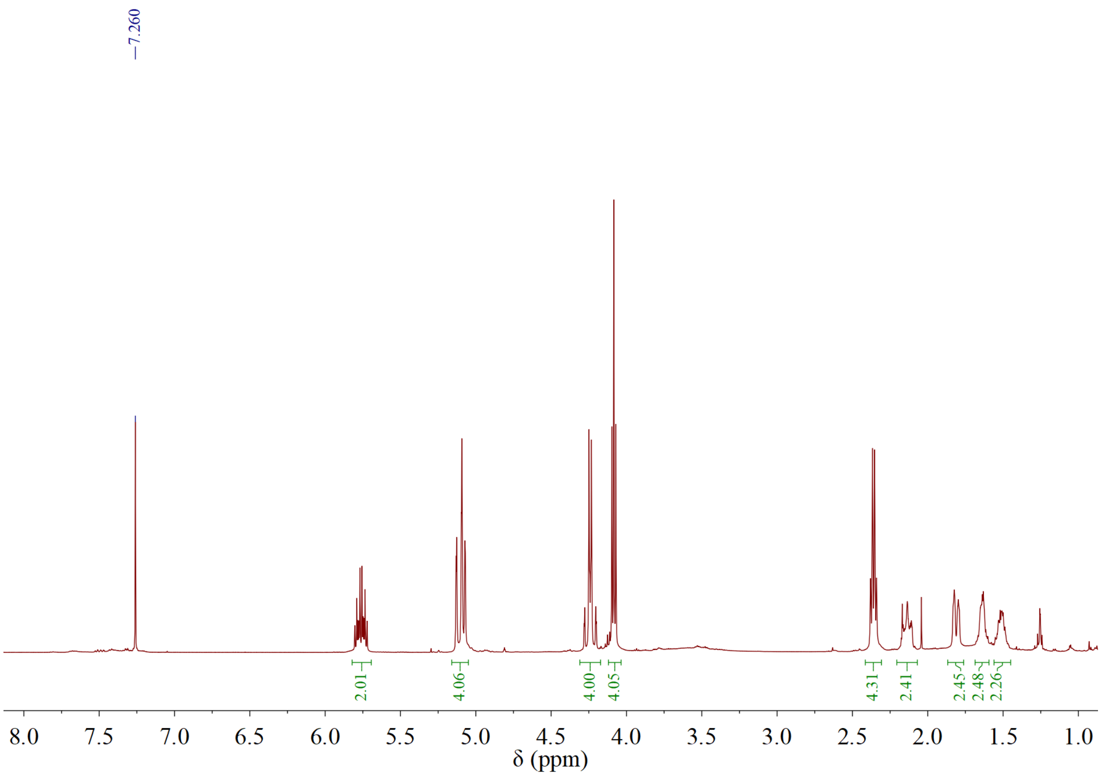

Supplementary Figure 33. ^{1}H NMR ($CDCl_3$, 500 MHz) spectrum of compound **2**.

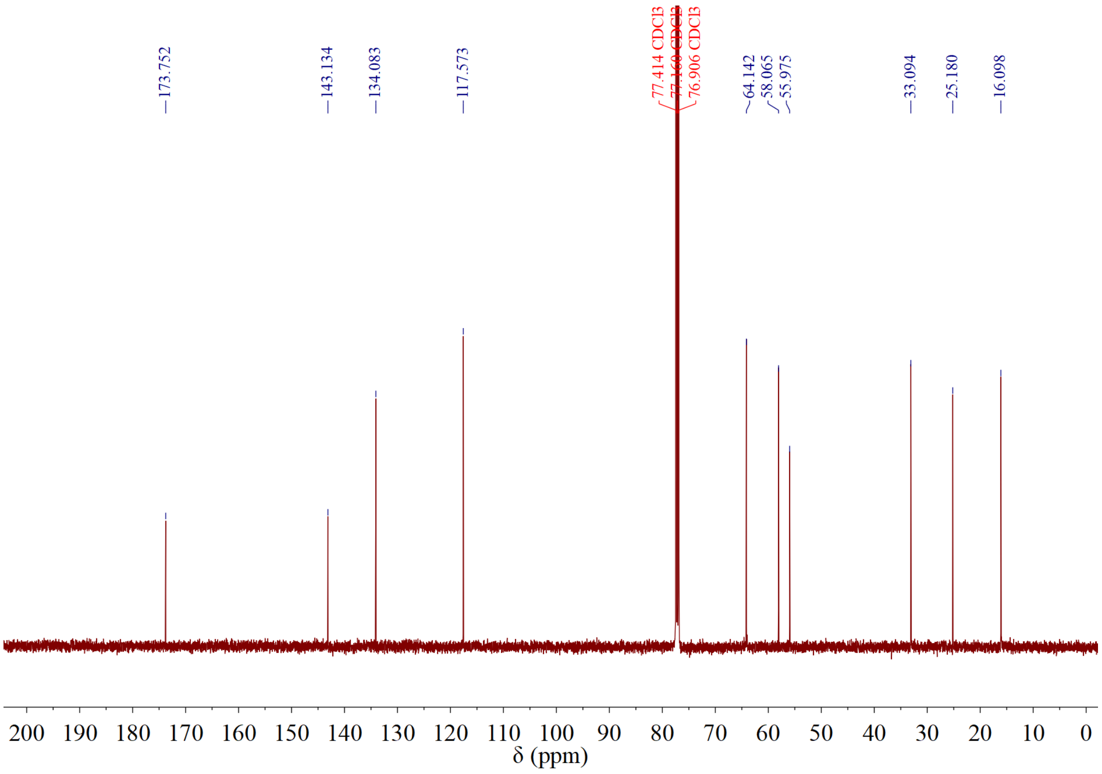

Supplementary Figure 34. ^{13}C NMR ($CDCl_3$, 125 MHz) spectrum of compound **2**.

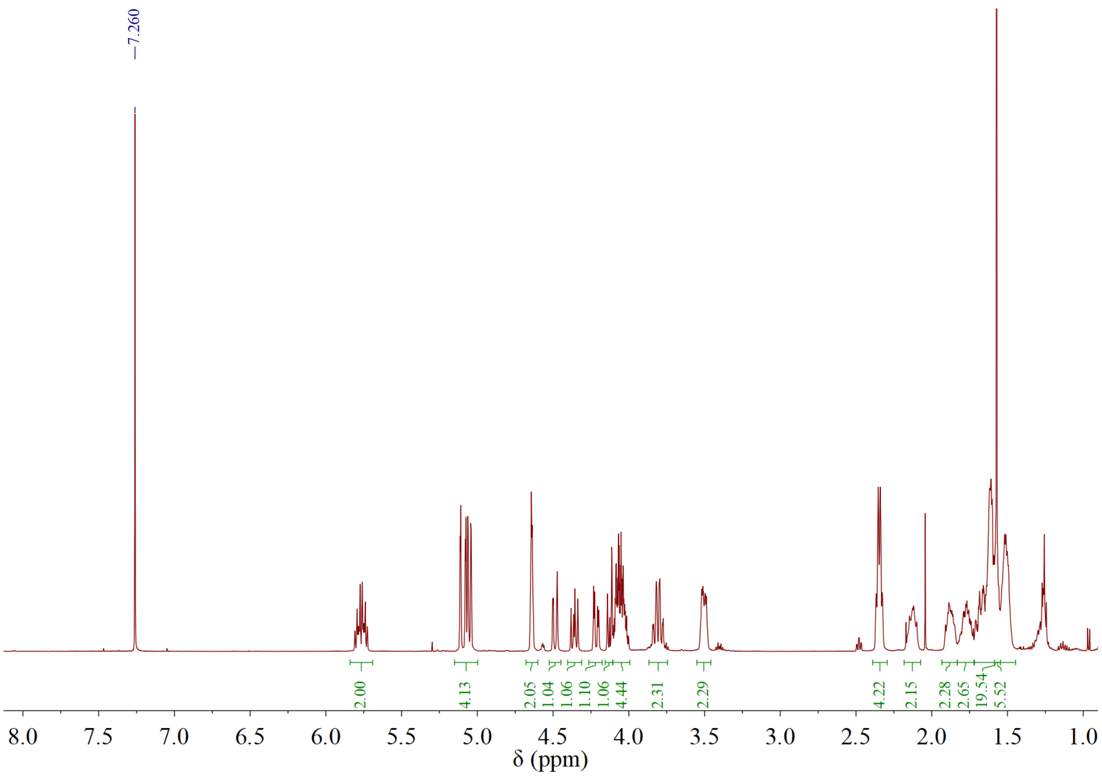

Supplementary Figure 35. ^{1}H NMR ($CDCl_3$, 500 MHz) spectrum of compound **3**.

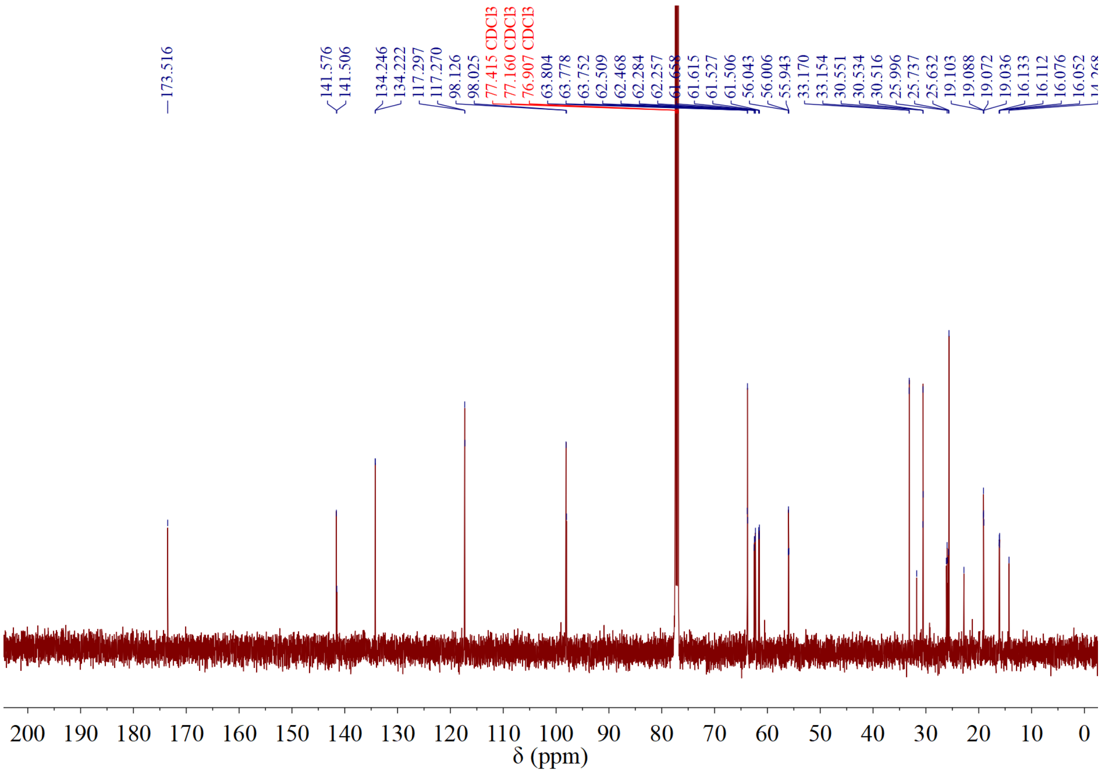

Supplementary Figure 36. ^{13}C NMR ($CDCl_3$, 125 MHz) spectrum of compound **3**.

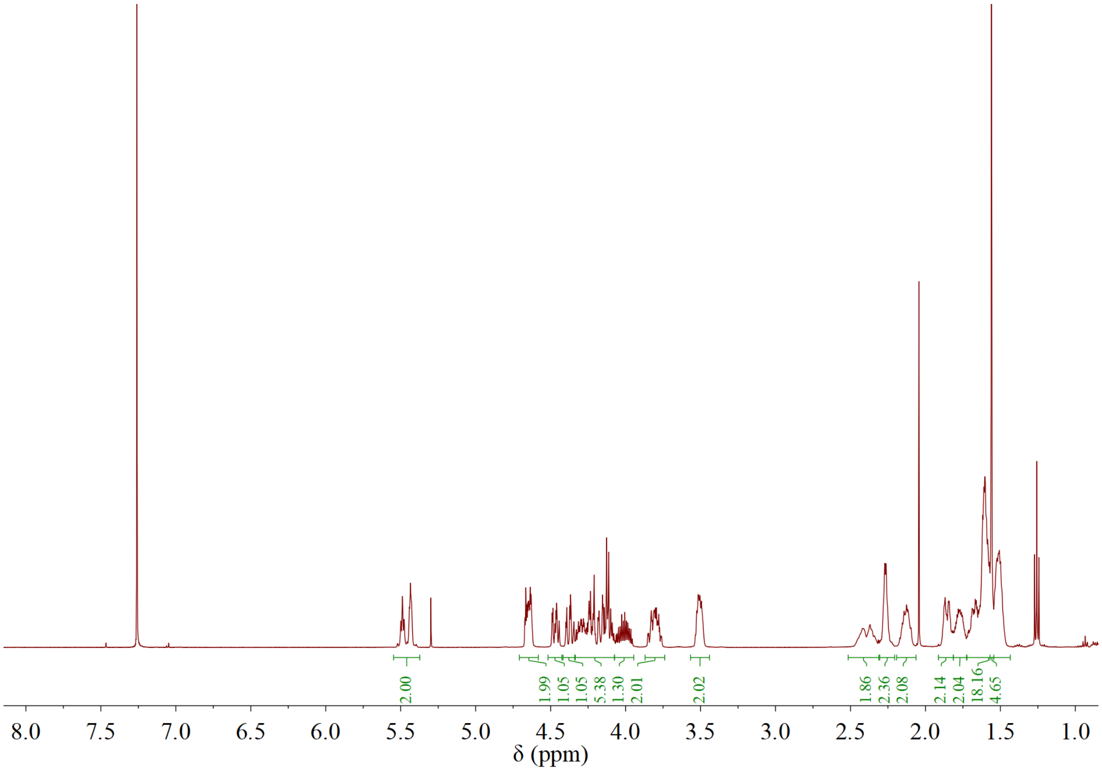

Supplementary Figure 37. ^{1}H NMR ($CDCl_3$, 500 MHz) spectrum of macrocycle **4**.

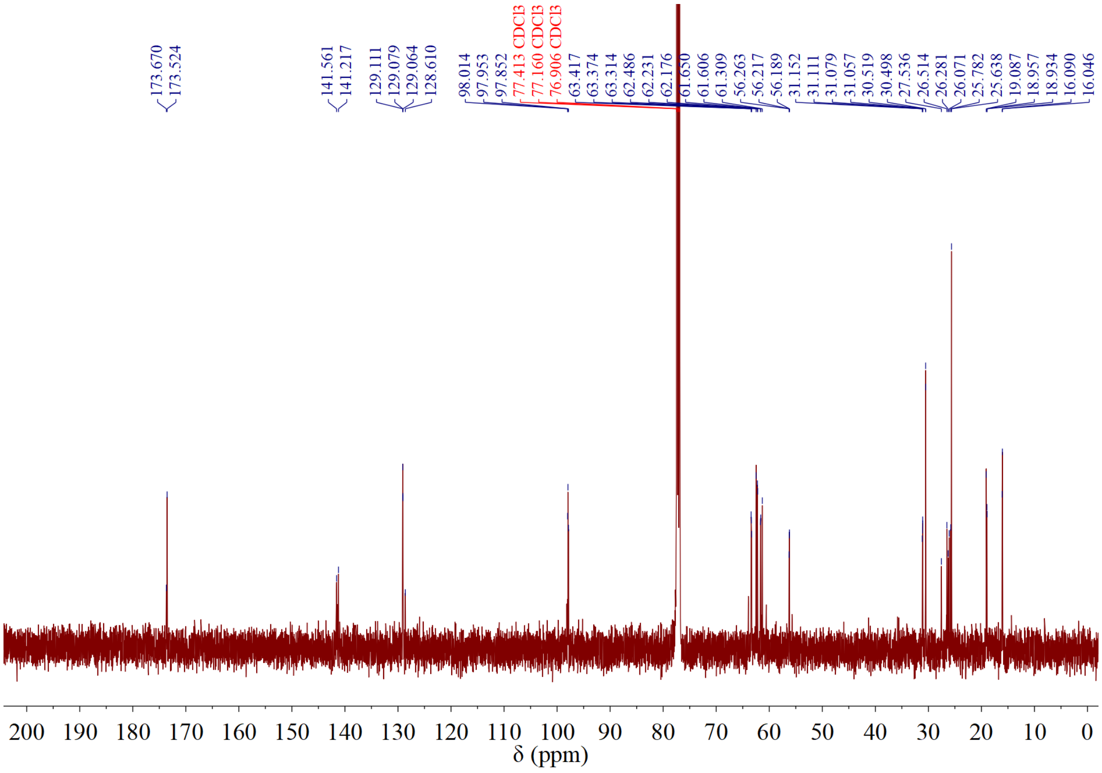

Supplementary Figure 38. ^{13}C NMR ($CDCl_3$, 125 MHz) spectrum of macrocycle **4**.

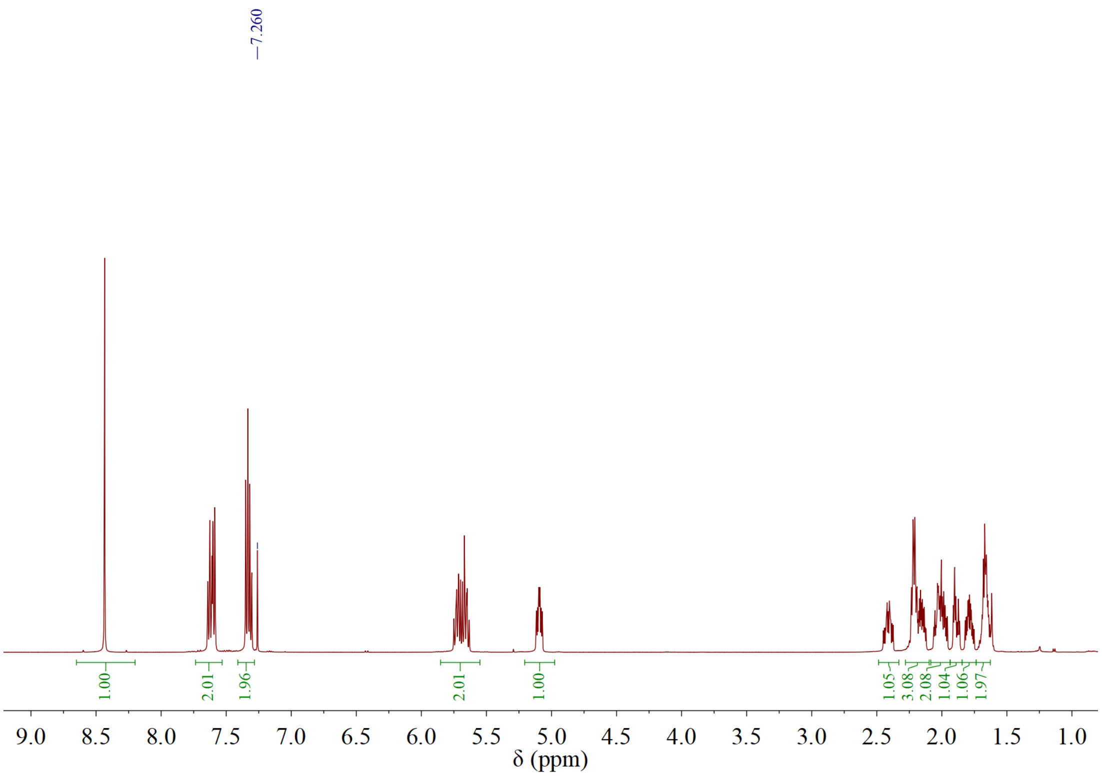

Supplementary Figure 39. ^{1}H NMR ($CDCl_3$, 500 MHz) spectrum of compound **5**.

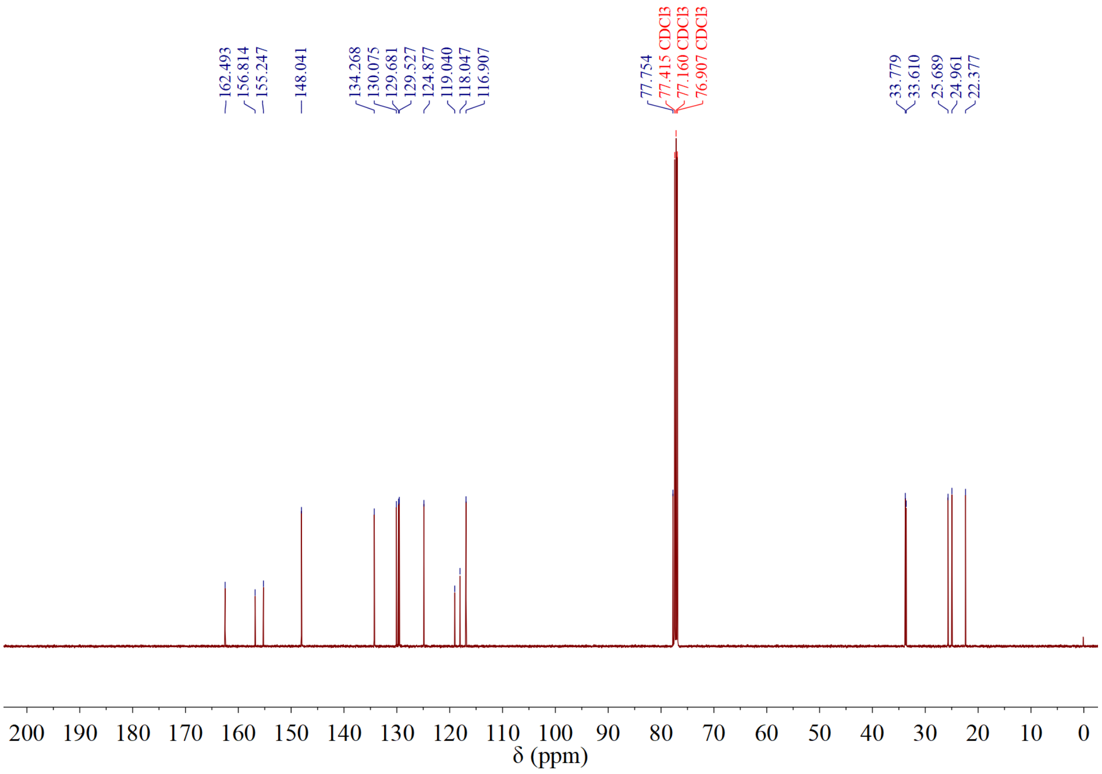

Supplementary Figure 40. ^{13}C NMR ($CDCl_3$, 125 MHz) spectrum of compound **5**.

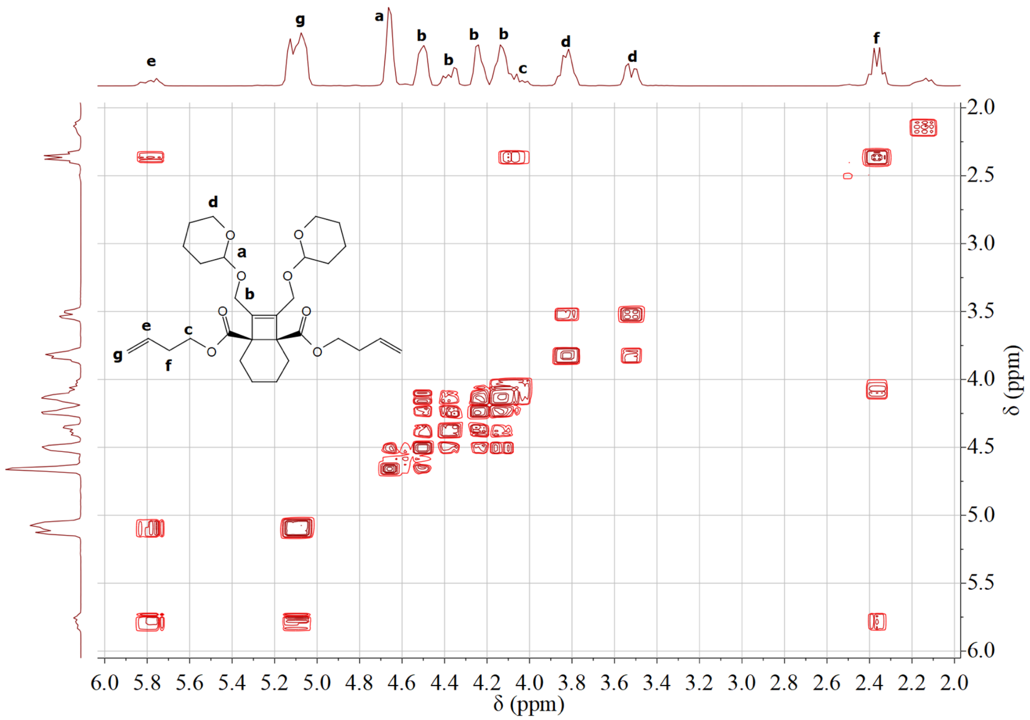

Supplementary Figure 41. COSY ($CDCl_3$, 500 MHz) analysis of compound **3**

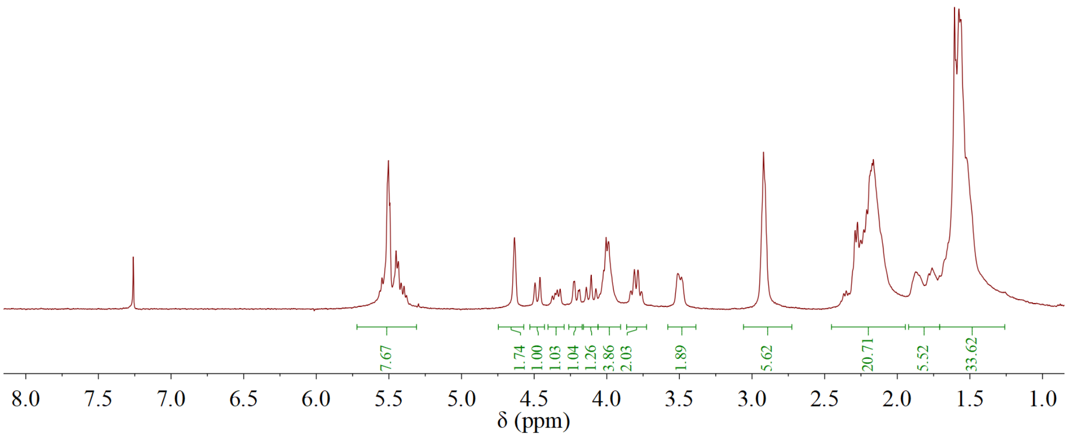

Supplementary Figure 42. ^{1}H NMR ($CDCl_3$, 500 MHz) spectrum of **P1**. BCOE content is: 1×2/7.67×100% = 26%

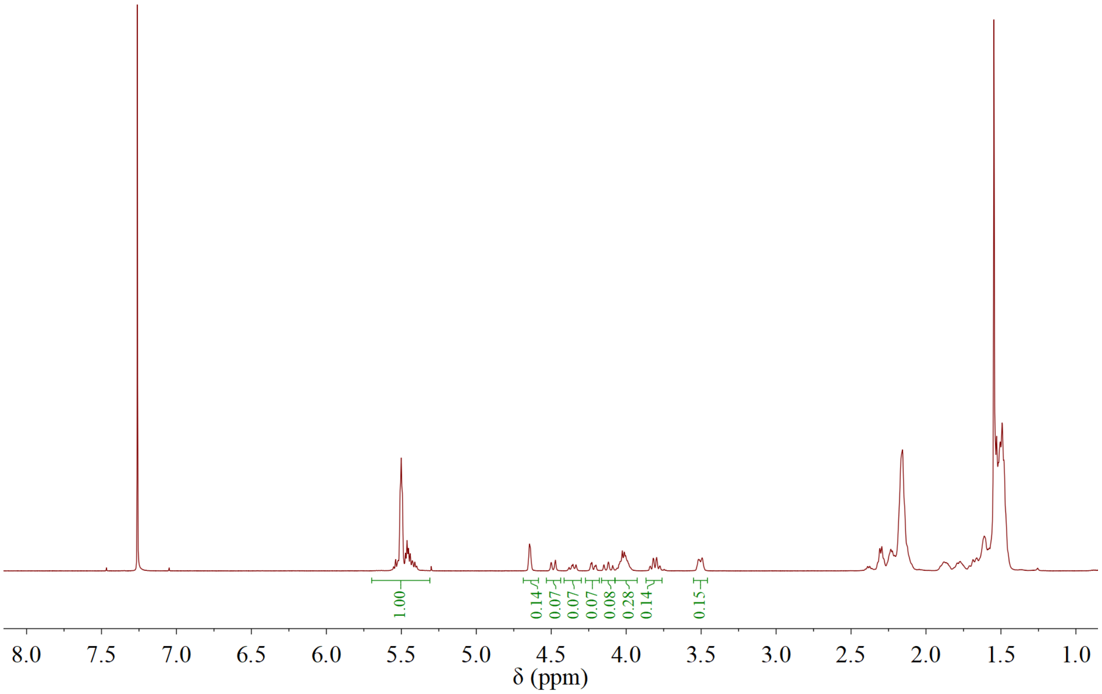

Supplementary Figure 43. ^{1}H NMR ($CDCl_3$, 500 MHz) spectrum of **P2**. BCOE content is: 0.07×2/1×100% = 14%

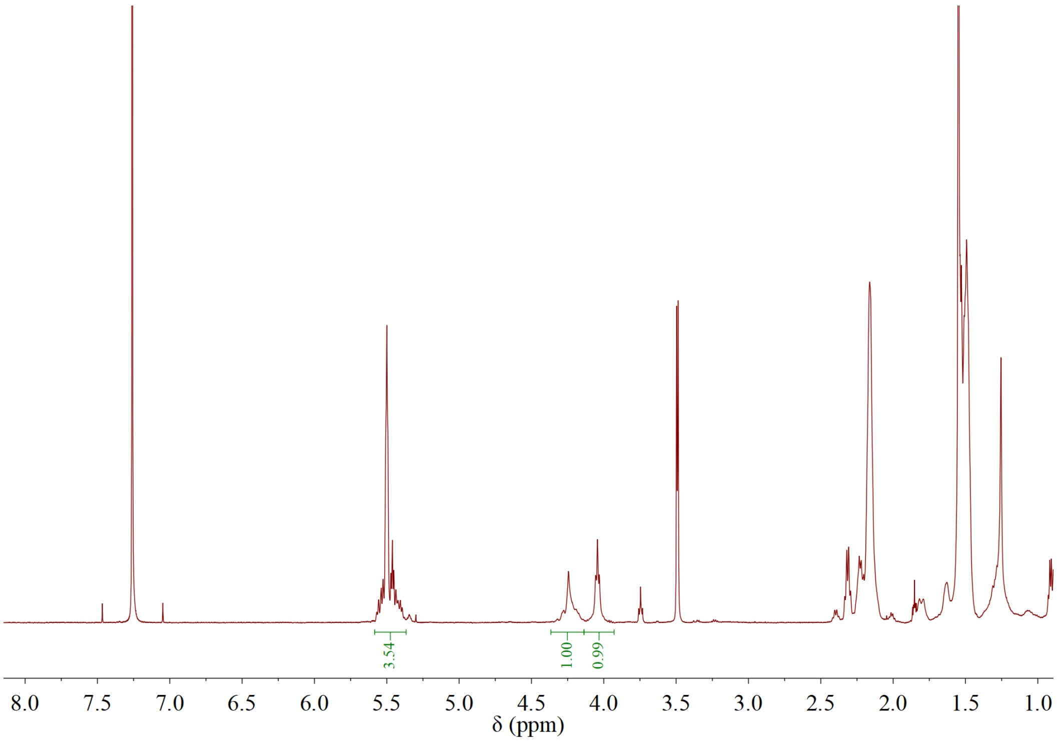

Supplementary Figure 44. ^{1}H NMR ($CDCl_3$, 500 MHz) spectrum of **P3**. BCOE content is: 1/(2×3.54)×100% = 14%

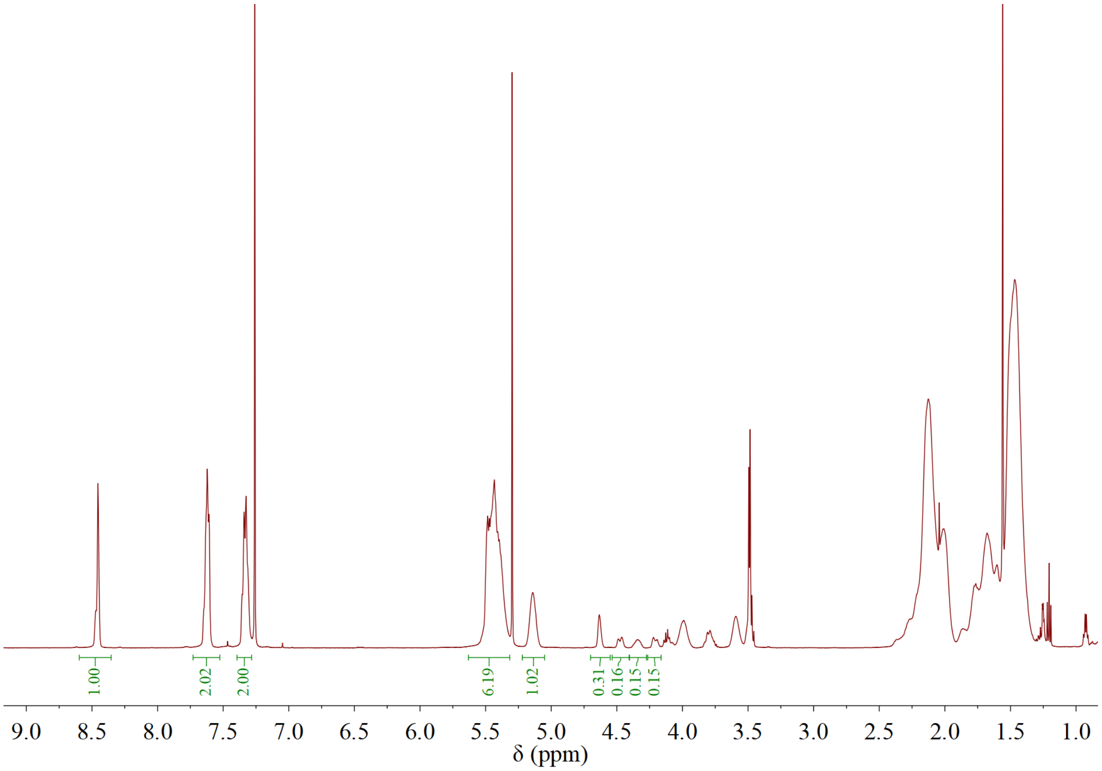

Supplementary Figure 45. ^{1}H NMR ($CDCl_3$, 500 MHz) spectrum of **P4**. BCOE content is: 0.31/6.19×100% = 5%

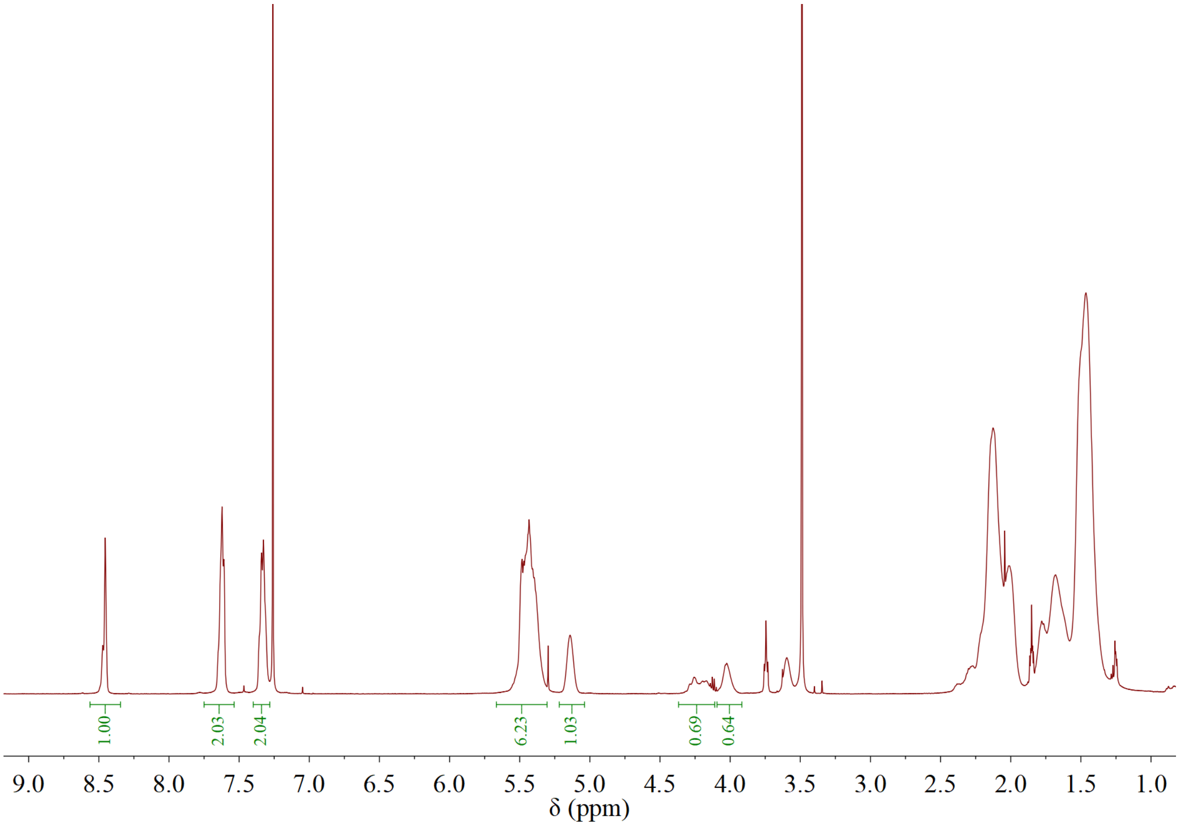

Supplementary Figure 46. ^{1}H NMR ($CDCl_3$, 500 MHz) spectrum of **P5**. BCOE content is: 0.64/2/6.23×100% = 5%

VII. Supplementary References

1. Klukovich, H. M.; Kouznetsova, T. B.; Kean, Z. S.; Lenhardt, J. M.; Craig, S. L. A Backbone Lever-Arm Effect Enhances Polymer Mechanochemistry. *Nat. Chem.* **2013,** *5*, 110-114.
2. Wang, J.; Kouznetsova, T. B.; Niu, Z.; Ong, M. T.; Klukovich, H. M.; Rheingold, A. L.; Martinez, T. J.; Craig, S. L. Inducing and Quantifying Forbidden Reactivity with Single-Molecule Polymer Mechanochemistry. *Nat. Chem.* **2015,** *7*, 323-327.
3. Booker-Milburn, K. I.; Cowell, J. K.; Harris, L. J. Model Studies Towards the Total Synthesis of Asteriscanolide. *Tetrahedron Lett.* **1994,** *35*, 3883-3886.
4. Booker-Milburn, K. I.; Cowell, J. K.; Sharpe, A.; Jiménez, F. D. Tetrahydrophthalic Anhydride and Imide: Remarkably Efficient Partners in Photochemical [2 + 2] Cycloaddition Reactions with Alkenols and Alkynols. *Chem. Commun.* **1996**, 249-251.
5. Booker-Milburn, K. I.; Cowell, J. K.; Harris, L. J. A Concise Synthesis of 7-Desmethylasteriscanolide and the Discovery of an Unusual Fragmentation Reaction to the Related Asteriscunolide Skeleton. *Tetrahedron* **1997,** *53*, 12319-12338.
6. Booker-Milburn, K. I.; Cowell, J. K.; Delgado Jiménez, F.; Sharpe, A.; White, A. J. Stereoselective Intermolecular [2+2] Photocycloaddition Reactions of Tetrahydrophthalic Anhydride and Derivatives with Alkenols and Alkynols. *Tetrahedron* **1999,** *55*, 5875-5888.
7. Neal, J. A.; Mozhdehi, D.; Guan, Z. Enhancing Mechanical Performance of a Covalent Self-Healing Material by Sacrificial Noncovalent Bonds. *J. Am. Chem. Soc.* **2015,** *137*, 4846-4850.
8. Lee, B.; Niu, Z.; Wang, J.; Slebodnick, C.; Craig, S. L. Relative Mechanical Strengths of Weak Bonds in Sonochemical Polymer Mechanochemistry. *J. Am. Chem. Soc.* **2015,** *137*, 10826-10832.
9. Sha, Y.; Zhang, Y. D.; Xu, E. H.; Wang, Z.; Zhu, T. Y.; Craig, S. L.; Tang, C. B. Quantitative and Mechanistic Mechanochemistry in Ferrocene Dissociation. *ACS Macro. Lett.* **2018,** *7*, 1174-1179.
10. Lin, Y.; Zhang, Y.; Wang, Z.; Craig, S. L. Dynamic Memory Effects in the Mechanochemistry of Cyclic Polymers. *J. Am. Chem. Soc.* **2019,** *141*, 10943-10947.
11. Black Ramirez, A. L.; Ogle, J. W.; Schmitt, A. L.; Lenhardt, J. M.; Cashion, M. P.; Mahanthappa, M. K.; Craig, S. L. Microstructure of Copolymers Formed by the Reagentless, Mechanochemical Remodeling of Homopolymers Via Pulsed Ultrasound. *ACS Macro. Lett.* **2011,** *1*, 23-27.
12. Booker-Milburn, K. I.; Delgado Jiménez, F.; Sharpe, A. Sequential Ring-Opening/Cyclisation Reactions of Bicyclo[4.2.0]Oct-7-Enes for the Synthesis of Cyclooctadiene Fused Lactones: Model Studies Towards the Total Synthesis of Pachylactone. *Tetrahedron* **1999,** *55*, 5889-5902.
13. Ralph, M. J.; Harrowven, D. C.; Gaulier, S.; Ng, S.; Booker-Milburn, K. I. The Profound Effect of the Ring Size in the Electrocyclic Opening of Cyclobutene-Fused Bicyclic Systems. *Angew Chem Int Ed Engl* **2015,** *54*, 1527-1531.
14. Wang, J.; Kouznetsova, T. B.; Craig, S. L. Reactivity and Mechanism of a Mechanically Activated Anti-Woodward-Hoffmann-Depuy Reaction. *J. Am. Chem. Soc.* **2015,** *137*, 11554-11557.
15. Wu D; Lenhardt, J. M.; Black, A. L.; Akhremitchev, B. B.; Craig, S. L. Molecular Stress Relief through a Force-Induced Irreversible Extension in Polymer Contour Length. *J. Am. Chem. Soc.* **2010,** *132*, 15936–15938.
16. Kouznetsova, T. B.; Wang, J.; Craig, S. L. Combined Constant-Force and Constant-Velocity Single-Molecule Force Spectroscopy of the Conrotatory Ring Opening Reaction of Benzocyclobutene. *Chemphyschem* **2017,** *18*, 1486-1489.
17. Lin, Y.; Kouznetsova, T. B.; Craig, S. L. A Latent Mechanoacid for Time-Stamped Mechanochromism and Chemical Signaling in Polymeric Materials. *J. Am. Chem. Soc.* **2020,** *142*, 99-103.
